# Spatial transcriptomics reveals brain-wide circadian disruption in an Alzheimer’s disease model

**DOI:** 10.64898/2026.01.26.701799

**Published:** 2026-01-28

**Authors:** Alon Gelber, Haylie Romero, Dominic Burrows, Daniel S. Whittaker, Daniel Carlin, Eran A. Mukamel, Paula Desplats

**Affiliations:** 1 Department of Bioengineering, University of California San Diego; La Jolla, United States.; 2 Department of Neuroscience, University of California San Diego; La Jolla, United States.; 3 Department of Cognitive Science, University of California San Diego; La Jolla, United States.; 4 Center for Circadian Biology, University of California San Diego; La Jolla, United States.

## Abstract

Diurnal rhythms in brain transcription align neural, immune, and metabolic processes with the light–dark cycle and are profoundly disrupted in Alzheimer’s disease (AD). However, the regional organization of diurnal transcription in the healthy and diseased brain remains poorly defined. Using large-scale spatial transcriptomics, we mapped 24-hour rhythmic transcription across cortical and subcortical regions of the mouse brain. We identified marked regional differences in rhythmicity, including distinct oscillatory signatures across cortical areas and along the rostro–caudal axis. In the APP23 mouse model of AD, pathology-vulnerable brain regions exhibited early, region-specific disruption of diurnal transcription prior to substantial amyloid plaque deposition. These findings reveal a spatially organized architecture of brain diurnal rhythms and identify early rhythmic dysregulation as a feature of Alzheimer’s disease pathogenesis.

Circadian rhythms are crucial regulators of brain function, rhythmic gene expression, synaptic plasticity, and metabolic cycles (1). Precise temporal regulation of these oscillations is essential for processes underlying learning, memory consolidation, and executive function(1). Spatial mapping of neural activity across the mouse brain reveals region-specific regulation of the phase and amplitude of circadian rhythms(2). Yet, previous studies have not systematically mapped spatially-resolved patterns of rhythmic brain transcription(3–7).

Mounting evidence indicates that disruption of intrinsic rhythmicity is a significant driver of cognitive decline and neurodegeneration(8–10). Disturbances of daily rhythms are prominent and debilitating symptoms affecting most Alzheimer’s disease (AD) patients, and include dysregulation of sleep/wake cycles(11) and exacerbation of cognitive impairment and confusion during the evening (known as “sundowning”)(12). Mechanistically, the circadian clock directly influences amyloid-β (Aβ) regulation and plaque formation (13–15). Rhythmic DNA methylation of the core clock gene *BMAL1*, (Basic helix-loop-helix ARNT-like protein 1), is altered in AD patient brains and fibroblasts and correlates with altered rhythmic transcription(16). These findings support the hypothesis that disruptions to the circadian system can drive neurodegeneration (9, 10).

Despite intensive research, the role of rhythmic gene expression in specific brain regions, and throughout disease progression remains unknown. This gap persists due to the lack of transcriptome-wide, spatially resolved information about the brain-region specific regulation of gene expression in controlled models of AD pathology. Here, we applied spatial transcriptomics (ST) to investigate diurnal gene expression cycles in the mouse brain and to identify alterations in rhythmic expression at early and advanced stages of neurodegeneration in a rodent model of AD(17–19). Our data represent a unique resource comprising 65 deeply sequenced mouse brains, covering 23 regions, 4 Zeitgeber time points, and two adult ages in both, non-transgenic controls and APP23-TG mice. We identified profound regional differences in rhythmicity, including distinct oscillatory signatures in the visual cortex compared to somatosensory, motor, and frontal areas. We detected thousands of differentially rhythmic genes whose diurnal expression patterns are disrupted in APP23-TG mice, particularly in brain regions highly vulnerable to AD pathology such as the dentate gyrus and cortex. Notably, rhythmicity was severely disrupted at early disease stages, prior to the overt Aβ pathology. These results suggest that brain-wide disruptions in rhythmic gene expression contribute to AD pathogenesis and may be targeted for clinical intervention.

## Results

### A spatial transcriptomic atlas of rhythmic gene expression across the mouse brain

**Fig. 1.**
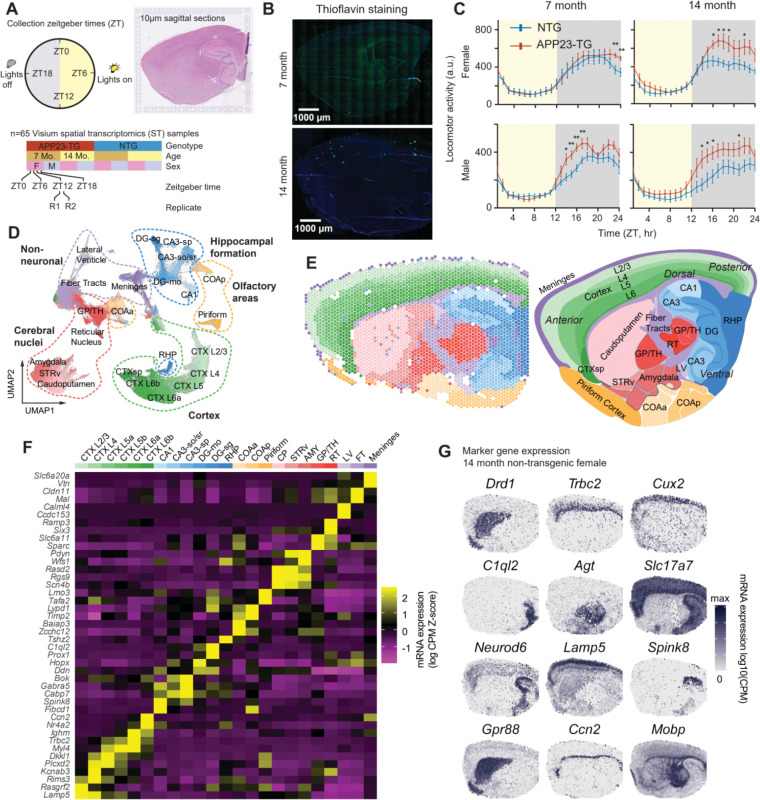
ST analysis of rhythmic gene expression across mouse brain regions and disease stages in the APP23 model of AD. **A,** Sequential 10-μm sagittal sections of APP23 transgenic (APP23-TG) and nontransgenic (NTG) brains were collected at 7 and 14 months of age, at four time points, including lights on and every six hours after, following monitoring of daily activity rhythms. **B,** The first section was used for ST and the next adjacent sections were used for Thioflavin S staining of plaques. **C,** Mean hourly activity under a 12 h light:12 h dark (LD) cycle at 7 and 14 months; mean ± SEM. Asterisks indicate significant genotype differences at Zeitgeber times (t-test; *p < 0.05, **p < 0.01). **D,** Embedding of 211,899 spots colored by annotated PRECAST clusters (n=65 samples). **E,** Clusters plotted on a representative slice and the Allen reference. **F,** Heatmap showing relative expression of top marker genes for each cluster. Row z-scored Log2 (count per million (CPM) +1). **G,** Examples of spatial expression of marker genes in a 14-month old NTG female.

We used spatial transcriptomics (ST) to comprehensively map rhythmic dynamics of gene expression across multiple brain regions (18, 20). We designed our study using Visium ST to take advantage of the fine-grained spatial resolution (50 μm spot diameter, 100 μm center-to-center spacing) and wide field of view capturing an entire sagittal mouse brain section (Fig. 1A).

To detect diurnal rhythms, we maintained mice in a 12:12 light dark cycle and collected brain samples at four Zeitgeber time points (ZT) every six hours from the lights on time (ZT0) (Fig. 1A). We applied this paradigm to male and female APP23 transgenic(21) (APP23-TG) and non-transgenic (NTG) littermate control mice at two key ages: 7 months, representing early-symptomatic mice, and 14 months, when transgenic mice show significant amyloid plaque burden. From each mouse, we collected a 10 μm thick section for ST and an adjacent 10 μm thick section that we stained for Thioflavin S to identify Aβ plaques (Fig. 1B). This data resource represents an unprecedented, comprehensive spatial map of brain diurnal gene expression.

The spatial resolution of the ST platform enables capturing mRNA from 1–10 cells, depending on the local cell density. We used spatially-aware clustering to define groups of ST spots based on similar gene expression and spatial contiguity(22). We chose cluster analysis parameters to match the anatomic and transcriptomic resolution of the Allen Brain Mouse Reference Atlas at the subclass level(23).

Our ST data provide comprehensive coverage of the mouse brain transcriptome at 211,899 total ST spots with an average of 15,696 unique transcripts (4,126 unique genes) detected per spot. We grouped these data into 23 anatomically and molecularly defined clusters (Fig. 1D–F). Cortical layers were separated based on layer-specific excitatory neuron markers, as were the major hippocampal subfields and layers (CA3 stratum radiatum (sr) and stratum pyramidale (sp); CA1; dentate gyrus (DG) molecular layer (mo) and stratum granulare (sg)) and retrohippocampus. We also identified major subcortical clusters including anterior and posterior cortical amygdala (marked by *Baiap3, Zcchc12* and *Lypd1*)(24), basal ganglia (cerebral nuclei) subregions such as reticular nucleus (*Ramp3)* and globus pallidus (*Sparc*), and piriform cortex (*Lmo3*, *Tafa2*). Finally, we identified clusters corresponding to white matter (*Mal*), ventricles (*Calml4*), and/or meninges (*Vtn)*. We annotated each cluster based on the expression of marker genes (Fig. 1F,G).

### Brain-region-specific rhythms of gene expression

Our experimental design enables testing rhythmic gene expression in the context of multiple variabless, including sex, age and genotype. We developed a flexible framework to detect 24-hour rhythmicity of gene expression using negative binomial harmonic regression of pseudobulk profiles with DESeq2(25). The model includes a rhythmic component expressed as a sinusoidal function of zeitgeber time (ZT). For each gene, we tested the significance of diurnal rhythmicity using a likelihood ratio test against a reduced model with no dependence on ZT. We validated that our approach is consistent with widely-used methods from the Metacycle package(26), while ours accommodates key covariates (Fig. S1A–D).

**Fig. 2.**
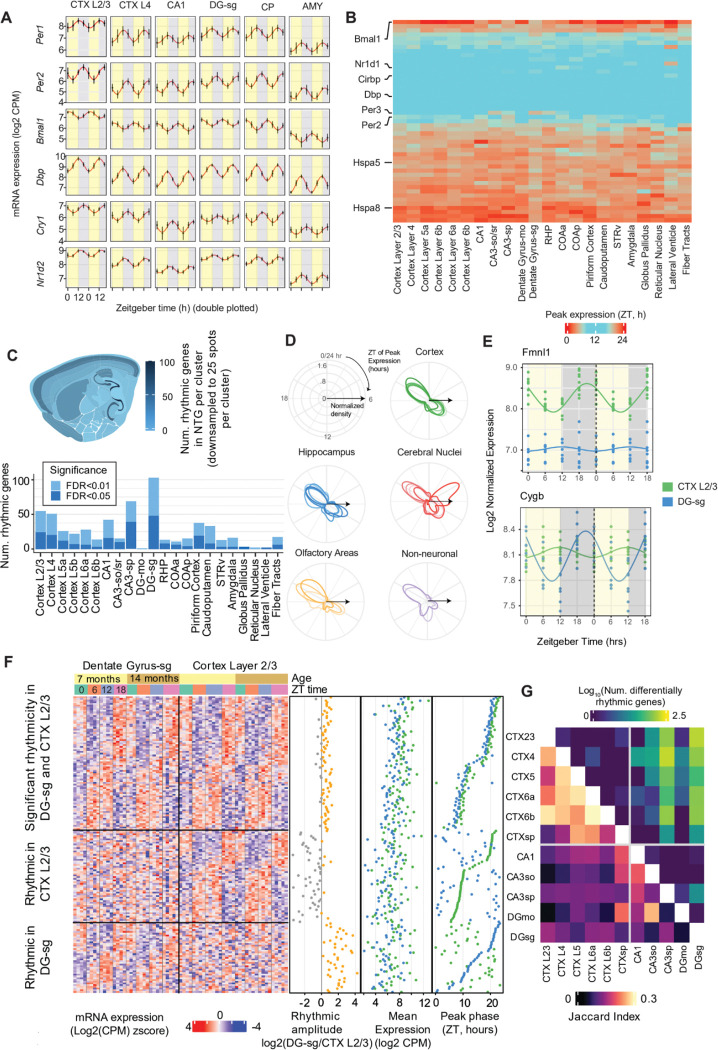
Spatial transcriptomic profiling of 24-hour rhythmicity in gene expression across the brain. **A,** Expression of core clock genes across brain regions. Data are double-plotted with sinusoidal model fit (red). Points and error bars show mean ± SD. **B,** Consistent phase of expression for the top rhythmic genes shared across brain regions (FDR<0.1 in at least 15 regions). **C,** Number of significantly rhythmic genes in each brain region in NTG mice (FDR<0.05, likelihood ratio test). Each cluster was downsampled to 25 spots. **D,** Polar plots of the distribution of peak phases for rhythmic genes. **E,** Region-specific rhythmic genes in cortex layer 2/3 (*Fmnl1*, green) and the dentate gyrus (*Cygb*, blue). **F,** Expression of rhythmic genes shared between cortex layer 2/3 and the dentate gyrus and region-specific rhythmic genes (DRGs, FDR<0.1 likelihood ratio test). **G,** Overlap of rhythmic genes and number of DRGs (LRT FDR<0.1) for all pairs of hippocampal and cortical clusters.

The circadian clock is defined by a conserved core oscillating circuit comprising *Per1, Per2, Bmal1 (Arntl)*, *Dbp, Cry1* and *Nr1d2(27)*. This circuit oscillates autonomously with a ~24-hour period in most tissues, including the brain, and is entrained to the ambient day-night cycle via light-activated neurons in the supra-chiasmatic nucleus (SCN) of the hypothalamus(28). The core molecular clock regulates a range of tissue- and cell type-specific effector genes in sync with the diurnal cycle to optimize cellular physiology to tissue-specific requirements. In the mouse brain, we found that core clock genes were significantly rhythmic in most brain regions (FDR<0.05, relative amplitude>0.05; Fig. 2A). The amplitude and phase of each core clock gene, defined by the ZT of peak expression (acrophase), were consistent across the brain (Fig. 2A,B). We found 119 rhythmic genes (FDR<0.2) in at least 10 out of 23 brain regions, and 52 genes were rhythmic (FDR<0.05) in ≥15 regions (Fig. 2B, Supplementary Table S1).

The number of significantly rhythmic genes varied across brain regions (FDR<0.05, Supplementary Table S2). To compare brain regions without bias due to differences in size, we downsampled the data from each region to 25 randomly selected ST spots per sample (Fig. 2C). Hippocampal and cortical regions had the largest numbers of significantly rhythmic genes (FDR<0.05). The granule cell layer of the dentate gyrus (DG-sg) had the most rhythmic genes (142 genes downsampled, 552 full dataset), followed by CA3sp (101 genes downsampled, 357 full dataset) and CA1 (57 genes downsampled, 172 full dataset) of the hippocampus. The cortex was also highly rhythmic, especially the upper layer 2/3 (63 genes downsampled, 1047 full dataset). Deep cortical layers, piriform cortex, and the caudoputamen had a moderate number of rhythmic genes, while cerebral nuclei and non-neuronal areas had the fewest (Fig. S2A).

The peak phase of rhythmic genes is largely consistent across tissues in humans(29), but differences in phase between brain regions have not been characterized. We found that the phase of rhythmic gene expression was bimodally distributed in the mouse brain, with most genes peaking around ZT8 or ZT20 hours (Fig. 2D). An exception was the reticular nucleus of the thalamus (RT), where 46 rhythmic genes peaked around ZT4. The RT serves as a relay between thalamic and cortical circuits and plays a critical role in sleep maintenance(30). Because non-rapid eye movement (NREM) sleep patterns are driven by reciprocal thalamic-cortical coupling, the unique phase profile of the RT may reflect its role in coordinating sleep stages(31). This finding highlights the importance of spatially resolved analysis of rhythmic gene expression, particularly for small but critical structures like the RT.

Our data allowed us to identify genes that have a brain-region specific rhythmic expression profile, potentially due to regional differences in diurnal activity(3). We found examples of genes with similar mesor levels across regions, but showing significantly different amplitude and/or phase of diurnal rhythmicity. To illustrate, we compared two regions with the highest number of rhythmic genes (DG-sg and Cortex L2/3, Fig. 2F). These regions shared 232 rhythmic genes (FDR<0.1), but each also had specific sets of rhythmic genes in only one region (1051 genes in L2/3, 419 in DG-sg). For example, the cell structure regulator Formin-like 1 (*Fmnl1*) had a strong diurnal rhythm in Cortex L2/3, but no apparent rhythmicity in DG-sg (Fig. 2E). By contrast, the oxygen regulator cytoglobin (Cygb) was strongly rhythmic in DG-sg but not Cortex L2/3.

To assess differential rhythmicity between regions, we used a likelihood ratio test that compares a model with independent harmonic coefficients in each region to a null model with shared harmonic components (FDR<0.1, Supplementary Table S3). This test is sensitive to differences in either phase or amplitude. We found that the rhythmic transcriptome is relatively conserved across cortical layers, with a strongly overlapping set of rhythmic genes (Jaccard index=0.21–0.28) and only a handful differentially rhythmic genes (DRGs, Fig. 2G, Supplementary Fig. S2D). By contrast, hippocampal clusters shared fewer rhythmic genes and had substantial differential rhythmicity with cortical regions.

### Regional specialization of rhythmic gene expression across major spatial axes of the hippocampus and cortex

**Fig. 3.**
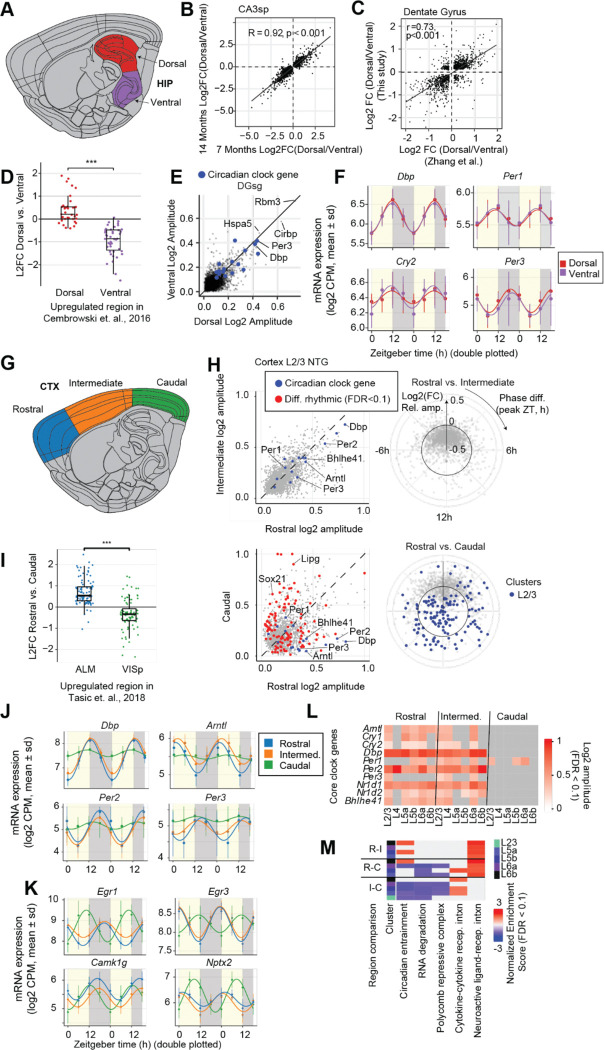
Dorso-ventral transcriptional gradients and subregion-specific rhythmicity across hippocampus and cortex. **A,** Hippocampal subregions**. B,** Dorsal/ventral log2 fold changes in representative hippocampal cluster in NTG mice at 7 versus 14 months (spearman r = 0.92, p < 0.001) **C,** Dorsal/ventral log2 fold changes in dentate gyrus comparing this dataset to Zhang et al. 2018 (32) bulk RNA-seq (r = 0.73, p < 0.001) **D,** Boxplot of dorsal vs. ventral log2 fold changes for genes annotated as dorsal or ventral markers in Hipposeq (Cembrowski et al. 2016(35)); points are individual genes (two-tailed t-test, ***p < 0.001). **E,** Scatter of log2 amplitudes in dorsal vs. ventral DG (FDR < 0.1); core clock genes highlighted. **F,** Rhythmic expression of representative transcripts in DG: points, mean ± SD at each ZT; curves, harmonic regression fits (dorsal, red; ventral, purple).**G,** Cortical subregions. **H,** Scatter of log2 rhythmic amplitudes in cortical layer 2/3 for rostral versus intermediate cortex (differential rhythmicity FDR < 0.1 in red); core clock (blue) genes highlighted. Inset polar plot depicts phase and amplitude differences. **I,** Boxplot of rostral vs. caudal log2 fold changes for genes annotated as ALM- or VISp-enriched (Tasic et al. 2018(34)); points are individual genes (two-tailed t-test, ***p < 0.001). **J,** Rhythmic expression of core clock genes across cortex (average over all layers): mean ± SD with harmonic regression fits for differentially rhythmic genes. **K,** Rhythmic expression for phase-shifted genes across cortex subregions; mean ± SD and harmonic fits. **L ,** Heatmap of log2 rhythmic amplitude for core clock genes (rhythmicity FDR < 0.1) across layers L2/3, L5a, L5b, L6a, L6b within the rostral, intermediate and caudal cortex. **M,** Heatmap of functional enrichment scores using GSEA for gene expression differences between cortical regions (R-I, rostral versus intermediate; R-C, rostral versus caudal; I-C, intermediate versus caudal) (FDR < 0.1).

Our ST dataset not only reveals distinct rhythmic regulation across major brain structures (Fig. 1E), but also resolves spatial differences within key structures. We leveraged this spatial resolution to compare the dorsal and ventral poles of the hippocampus (Fig. 3A) and cortical regions ranging from rostral motor and somatosensory areas to caudal visual cortex (Fig. 3G).

Thousands of genes showed distinct expression patterns between the dorsal and ventral poles of hippocampal areas, with the most DEGs observed in area CA3 (Fig. S3D, Supplementary Table S4). We confirmed previous findings of a robust dorsal-ventral gradient of expression of multiple marker genes, including patterning factors like *Epha7* and *Nr2f2* (Fig. S3D), as well as *Trhr* and *Lct* (Fig. S3E–F)(32, 33). The dorsal-ventral gradient of gene expression was consistent with previous studies using bulk RNA-seq from the dorsal and ventral poles of the DG(32) (Fig. 3C, Pearson r=0.73, p<0.001). We found that dorsal genes in all 4 hippocampal clusters were functional enriched for long-term potentiation (FDR<0.05, see Methods). Ventral-enriched genes had more variable functional profiles (Fig. S3H).

Despite the large transcriptional differences in mean expression between dorsal and ventral hippocampal regions, we found highly consistent rhythmic modulation in both poles of the hippocampus (Fig. 3H). Core clock genes showed nearly identical amplitude and phase of expression (Fig. 3H). No genes had significantly different rhythmicity in dorsal vs. ventral hippocampus (interaction between region and ZT, FDR<0.1). Thus, although dorsal and ventral hippocampal regions have robust differences in gene expression, their circadian regulation appears to be highly consistent.

The neocortex is organized along the rostro-caudal axis in major regions specialized for cognitive functions, from the primary visual cortex (VISp) at the caudal pole to somatosensory in middle (intermediate) and motor and frontal cortices at the rostral end. Single cell transcriptomics has identified hundreds of genes that are differentially expressed in VISp vs. antero-lateral motor (ALM) cortex(34). We found thousands of DE genes in pairwise comparisons of rostral vs. caudal, rostral vs. intermediate, and intermediate vs. caudal regions (Fig. 3H, FDR<0.05, Supplementary Table S5). Rostral vs. caudal DEGs were largely consistent with previous scRNA-seq from VISp and ALM(34) (Fig. 3I).

Caudal genes were significantly enriched in functional categories including “circadian entrainment” and “RNA degradation” (Fig. 3M). These genes included components of intracellular calcium signaling (*Adcyap1, Camk2d, Cacna1c/d/h*), MAPK signaling (*Mapk3*), activity-regulated genes (*Fos, Rasd1*), and other modulators of circadian input pathways (*Adcy8, Grin2b, Prkg2, Prkacb*) (Fig. 3M). Importantly, this analysis did not explicitly account for ZT and thus reflects regional differences in the mesor, or mean gene expression across the day. Our data thus suggest the caudal cortex, despite having less rhythmic expression of core clock genes, may be transcriptionally primed for circadian entrainment and activity-dependent signaling.

Furthermore, analysis of rhythmic transcription identified robust differences in gene expression in the caudal cortex compared to rostral and intermediate regions. There were up to 390 DRGs between caudal and rostral or intermediate cortex across cortical layers, while ≤3 genes were significant DRGs between rostral and intermediate cortical regions (Fig. 3H, FDR<0.1, Supplementary Table S6). This was notable, since there were hundreds of genes with significantly different expression mesor in rostral, intermediate and caudal cortical regions (Supplementary Fig. S3I).

Notably, the rhythmicity of several core clock components was attenuated or abolished in the caudal cortex. These included *Arntl (Bmal1), Nr1d1 (Rev-Erbα), Nr1d2 (Rev-Erbβ), Dbp, Per2,* and *Bhlhe41 (DEC2)*, which were substantially more rhythmic in rostral and intermediate compared with caudal cortex (Fig. 3J). The rhythmic amplitude of core clock genes was consistently lower in caudal regions across cortical layers (Fig. 3L). Gene ontology analysis showed that 177 DRGs with stronger rhythmicity in rostral compared with caudal cortex were enriched for circadian rhythm function (FDR<0.001, Supplementary Table S7).

We further identified a distinct set of 169 genes that were rhythmic in caudal cortex, but lacked significant rhythmicity in rostral or intermediate regions. These caudal-specific rhythmic genes were enriched for regulators of MAPK signaling, such as *Dusp5, Dusp6, Adcy8,* and *Elk1* (Fig. 3H). The robust diurnal rhythmicity of these genes shows that caudal cortex does not lack a 24-hour clock, despite the reduced rhythmicity of core clock components.

We identified 22 genes that had significant rhythmic expression in both caudal and rostral/intermediate cortex but with significant differences in phase, including *Egr1, Egr3, Camk1g*, and *Nptx2* (Fig. 3L). The phase shift between rostral vs. caudal regions was consistent across cortical layers. These genes are involved in activity-dependent transcriptional programs, synaptic signaling, and calcium-mediated pathways, and their coordinated phase shifts across layers suggest region-specific regulation of their rhythmic expression. For example, in nocturnally active species like mice, visual stimulation during the light phase could drive increased expression of neural activity-related genes in caudal cortex, whereas somatosensory and motor regions would be more active during the dark phase. These findings are consistent with a recent neural activity mapping by c-Fos protein expression, which also identified an opposite phase of circadian rhythmicity in visual cortex compared to other cortical regions(2). Finally, we confirmed that the within-region expression gradients and specific rhythmic patterns we observed in NTG animals were also present in APP23-TG mice, and thus seems spared from pathology-associated alterations (Supplementary Fig. S4).

### Disruptions of brain-region specific transcriptomes in APP23-TG AD mice

To investigate the impact of AD-related pathology on rhythmic gene expression across the brain, we performed ST in APP23-TG mice(9, 21, 36). Male and female APP23-TG mice had disrupted circadian behavioral patterns, fragmented sleep and increased locomotor activity in the home cage during the dark phase(9) (cage activity) (Supp. Fig. S5A,B), consistent with our previous report(9). APP23-TG mice show early circadian disruption at 7 months of age, despite the absence of overt amyloid pathology, with a longer intrinsic circadian period (free-running period) than NTG mice, measured in constant darkness, (p ≤ 0.05, Supplementary Fig. S5D). By contrast, at 14 months of age both APP23-TG and NTG animals had equivalent intrinsic circadian periods, which were longer than that of 7 month NTG animals.

Our findings are in line with the characterization of APP23-TG mice showing development of progressive neuropathology starting around 6 months of age with sparse Aβ buildup, followed by accumulation of plaques at 7–9 months in the cortex which then spread to the hippocampus(37) (Fig. 1B, Supplementary Fig. S6A,B). This was accompanied by increased expression of reactive astrocyte marker, GFAP (Supplementary Fig. S6C,D). As the disease progressed, both Aβ plaque burden and astrogliosis increased as did the development of microgliosis throughout the cortex (Supplementary Fig. S6E–G)(21).

**Fig. 4.**
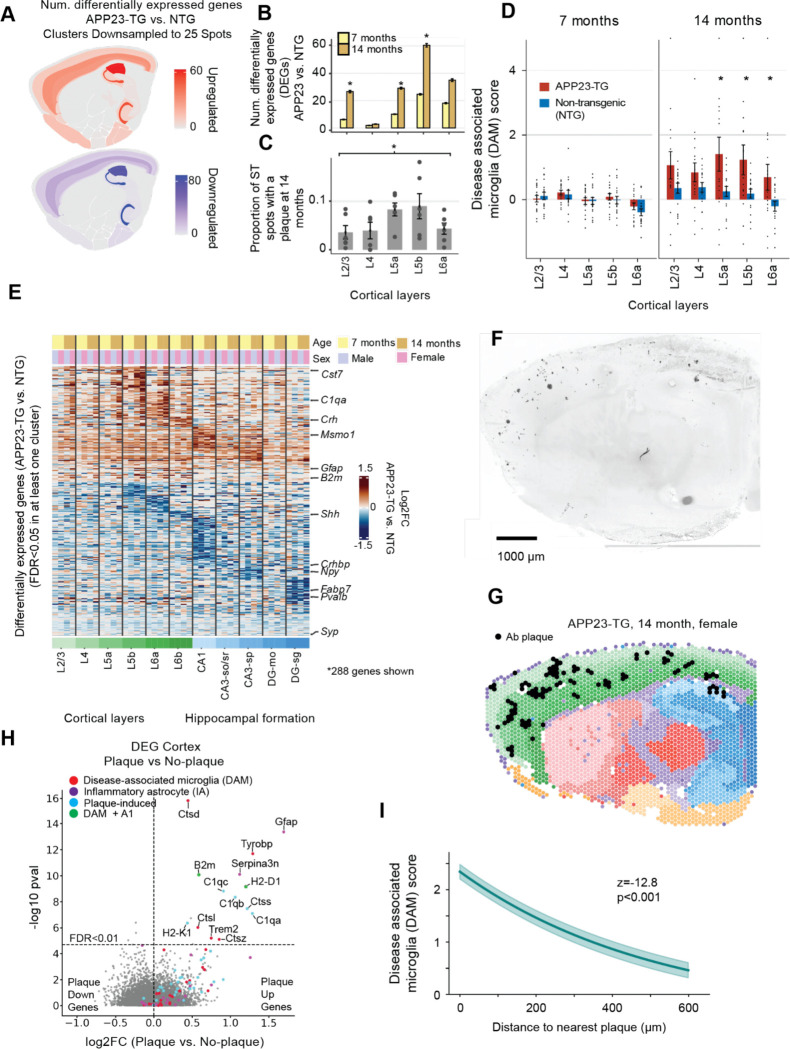
Altered spatial patterns of mRNA expression in APP23-TG mice. **A,** Number of differentially expressed genes (DEGs) in APP23-TG vs. NTG mice in each brain region (FDR<0.05) at 14 months. Clusters were downsampled to 25 spots per sample. **B,** Number of DEGs in APP23-TG mice per cortical layer at 7 and 14 months. DE analysis was performed by downsampling cortical layers to 100 spots 20 times and computing the mean and standard error of the number of DEGs with a Wald test (FDR<0.05). *: p<0.05 (Wilcoxon test, 7 vs. 14 months). **C,** Mean Aβ plaques per ST spot in each cortical layer in 14 month APP23-TG mice (errorbar: SEM; dots: individual samples). *: p<0.05, mixed model controlling for sex and sample as a random effect (p<0.05). **D,** Disease associated microglia (DAM) score for cortical layers (errorbar: SEM; dots: individual samples). *: p<0.05, T-test, APP23-TG vs NTG. **E,** 288 DEGs in cortex and hippocampus (FDR<0.05, LRT: ~sex+age+genotype vs. ~sex+age; mean(|L2FC|)>0.3, max(|L2FC|)>0.7). **F,** Example of Thioflavin S staining in 10 μm sections adjacent to sections used for ST. **G,** Example 14 month APP23-TG sample with annotated plaques. **H,** 88 DEGs between Aβ plaque-associated spots and non-plaque associated spots in the cortex (FDR<0.1, LRT: ~plaque+(1|sample) vs. (1|sample) ). Significantly DE DAM, inflammatory astrocyte (A1) and plaque-induced (PI) genes(43) are highlighted. **I,** Exponential fit of the average DAM score for each spot as a function of the distance to the nearest plaque. *908 genes with FDR<0.1 (LRT: ~plaque_distance+(1|sample) vs. (1|sample) ).

To determine whether AD pathology impacts brain region-specific gene expression, we identified differentially expressed genes (DEGs) between APP23-TG and NTG mice using conservative, statistically rigorous criteria (see Methods)(25, 38). We observed up to 498 DEGs per brain region, with the largest changes located in the hippocampus, dentate gyrus and cortex (FDR<0.1, Fig. 4A, Supplementary Table S8). In the cortex, the greatest number of DEGs was found in layer 5b in both the pre-amyloid (7 month old) and advanced pathology (14 month) groups (Fig. 4B). The distribution of DEGs across layers mirrored the Aβ plaque burden in 14-month old APP23-TG mice, as quantified by the proportion of plaque-associated spots per cortical layer (Fig. 4C).

In the cortex and hippocampus, DEGs included hallmarks of neuroinflammation in AD such as microglial genes *Cst7* and *B2m* (Fig. 4E). Neurodegeneration has been linked to an increased proportion of disease-associated microglia (DAM)(39). We created a gene expression-based DAM score using known markers of Alzheimer’s DAM(39–42). The DAM score was significantly higher in 14 month old APP23-TG mice compared with NTG controls, particularly in deep cortical layers (p<0.05, Fig. 4D).

### Genes associated with AD pathology are activated in the proximity of Aβ plaques in APP23-TG mice.

Aβ plaques can alter the regulation of nearby cells by inducing expression of genes involved in myelination, complement signaling, oxidative stress and inflammation(43). To address the spatial relationship between Aβ plaques and altered gene expression in our ST data, we used thioflavin S staining to identify plaque associated spots in brain sections immediately adjacent to those used for ST (Fig. S6C). The average number of Aβ plaques per sample increased from 7±6 at 7 months of age to 42±37 at 14 months in APP23-TG animals (mean ± SD, p<0.01, n=9; Fig. S6B).

To identify plaque-induced genes, we analyzed DEGs in plaque-associated vs. non-plaque-associated ST spots in the cortex using a mixed effects model to account for inter-sample variability. We found 47 upregulated and 41 downregulated DEGs in plaque-associated spots (FDR<0.1, Supplementary Table S9). The DEGs included known markers of disease pathology including DAM-associated, inflammatory astrocyte (A1) and other plaque-induced genes like *C1q(a-c)*, *Ctss* and *Npc2* (Fig. 4H). Notably, the plaque-induced genes overlapped with findings from another AD mouse model, App^NL-G-F^ (22 shared genes; p<0.0001 Fisher’s exact test)(43), validating the findings. Furthermore, we found that 11 key DAM genes had higher expression in spots neighboring a plaque compared with non-plaque associated spots (FDR<0.01, Fig. 4H). The DAM score was significantly higher in spots located <200 μm from a plaque compared to spots with no nearby Aβ plaques (Fig. 4I).

### Increased rhythmic amplitude of gene expression in APP23-TG mice

**Fig. 5.**
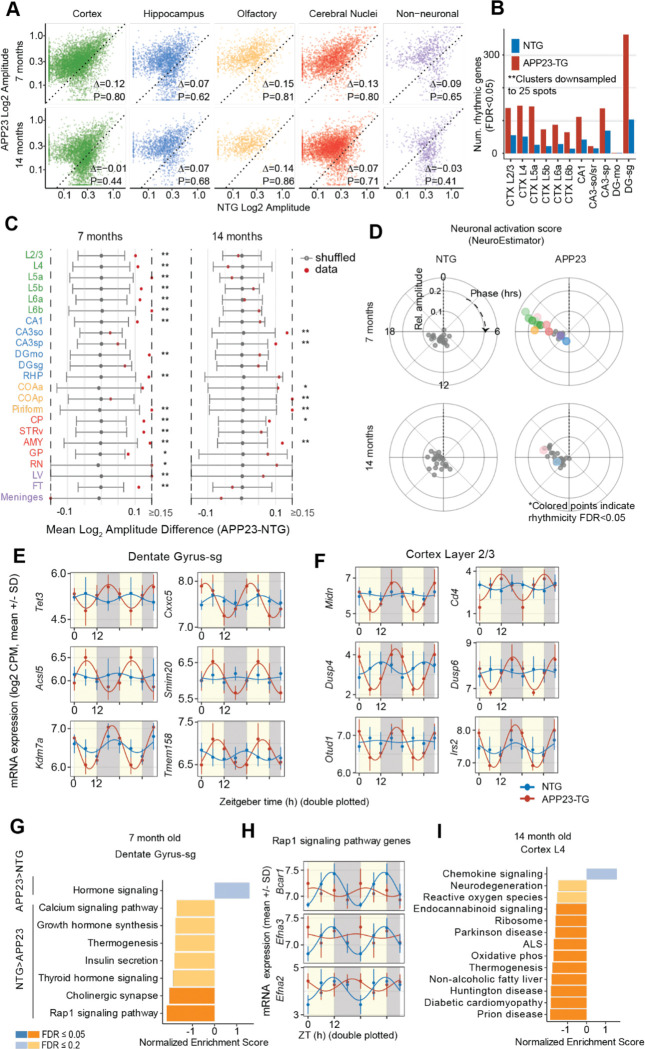
Alterations in diurnal rhythmicity of gene expression in APP23-Tg mouse brain. **A,** Log2 amplitude of rhythmic expression in APP23 vs. NTG (dashed line shows slope=1). **B,** Number of significantly rhythmic genes in APP23-TG and NTG (FDR<0.05) across cortical and hippocampal regions. **C,** Difference in mean amplitude of rhythmicity between APP23 and NTG for each cluster (red points) plotted on null distribution of the same metric from 500 random permutations of the genotype labels. Grey error bars show 95% confidence interval of the empirical null distribution from 500 random permutations of the genotype labels (*, FDR<0.05). **D,** Phase and amplitude of mean neuronal activation score(44) by cluster, age and genotype. Significantly rhythmic clusters (LRT FDR<0.05) are indicated by the colored points while non-rhythmic scores are gray. **E,** Expression of several example genes with greater rhythmicity in APP23-TG compared to NTG (interaction LRT FDR<0.05) in dentate gyrus. **F,** Same as previous in cortex layer 2/3 . **G,** Gene set enrichment analysis on log2 amplitude difference for rhythmic genes in dentate gyrus in 7 month-old animals(45, 46).**H,** Expression of three Rap1 signaling genes (Bcar1, Efna3, Efna2) in cortex layer 4 at 14 months with diminished rhythmicity in APP23-TG compared to NTG. **I,** Gene set enrichment analysis on log2 amplitude difference for rhythmic genes in cortex layer 4 in 14 month-old animals(45, 46).

Rhythmic gene expression is disrupted in the AD brain(8–10, 47, 48), but the regional pattern of disruption has not been established. We identified hundreds of genes that gained or lost rhythmicity in APP23-TG compared to NTG animals. Notably, we observed a greater number of rhythmic genes in APP23-TG animals across all brain regions compared to controls (Fig. 5B), and an increase in amplitude in most rhythmic genes brain-wide (Fig. 5A). To statistically test the difference in rhythmicity for individual genes, we used a conservative, permutation-based procedure to generate an empirical null distribution (See Methods, Fig. 5C). The increased rhythmic amplitude in APP23-TG mice was most pronounced in 7 month old animals, though it was still evident in 14 month old mice with more advanced pathology (Fig. 5C). Consistent with this global increase in rhythmic amplitude, hundreds of genes with minimal or undetectable rhythms in NTG controls acquired clear 24 h oscillations in 7-month-old APP23-TG mice. In the dentate gyrus, *Tet3*, a methylcytosine dioxygenase that initiates DNA demethylation(49), and *Cxxc5*, which antagonizes Wnt/β-catenin signaling and has been linked to AD(50), both became significantly rhythmic in APP23-TG (interaction LRT FDR < 0.05; Fig. 5E). In cortex layer 2/3, APP23-TG induces robust diurnal oscillations in ubiquitin regulators (*Midn*, *Otud1*, *Uxt*)(51) and phosphatases (*Dusp4*, *Dusp6*), with peak expression clustering around the transition from dark to light. The increased rhythmicity of protein quality control and signal‐termination pathways in cortex may represent an adaptation to early pathological changes (interaction LRT FDR < 0.05; Fig. 5F).

We found significant functional enrichment among genes with decreased rhythmic amplitude (Fig. 5G). Genes losing rhythmicity in dentate gyrus of 7-month-old APP23-TG animals, including *Bcar1, Efna2,* and *Efna3,* were enriched for pathways linked to AD, like Rap1 signaling, cholinergic synapse, insulin metabolism, thermogenesis, and calcium signaling, (FDR<0.05, Fig. 5G,H) (52, 53)(54, 55). On the other hand, genes that gained rhythmicity in APP23-TG were enriched for insulin secretion (e.g. *Irs2*) and parathyroid hormone signaling in cortex layer 2/3.

### Activity dependent genes gain rhythmicity at early stages of AD pathology

Daily rhythms in neural activity lead to oscillation of a set of immediate early genes (IEGs), as recently shown by immunostaining of c-Fos protein(2). We found that the genes that gained rhythmicity in APP23-TG cortex and dentate gyrus were enriched in neuronal activity-induced genes (FDR<0.05)(56). Because IEGs are rapidly induced by neural activity, shifts in their diurnal phase and/or amplitude suggest altered daily rhythms of cortical and hippocampal neural activity in APP23-TG.

To investigate this, we estimated neural activity scores based on the combined expression of a panel of activity-dependent genes(44). We compared these scores between APP23-TG and NTG mice at 7 and 14 months across brain regions using a linear mixed-effects model. We found no significant differences in the mesor of neural activity scores between the genotypes. Activity scores for 12 of the 23 clusters had a significant 24-h rhythm in 7-month-old APP23-TG mice, and in 2 clusters in 14-month-old APP23-TG mice (FDR<0.05, Fig. 5D). By contrast, NTG mice had no significant rhythmicity in neural activity scores (Fig. 5D). Using a likelihood ratio test, we established that APP23-TG mice had significantly higher rhythmicity of the activity score than NTG controls in all cortical clusters and in the DG at 7 months (FDR<0.05). These findings suggest early amyloid pathology may drive synchronized and phase-shifted neuronal activation across the brain.

### Brain region-specific phase shifts in APP23-TG mice

**Figure 6.**
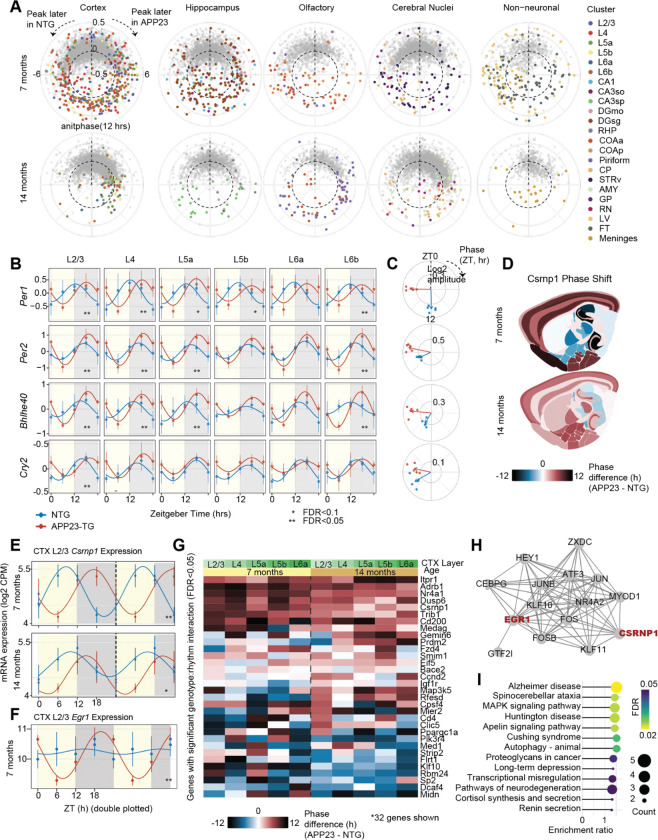
Phase dysregulation of rhythmic gene expression in APP23-TG varies with disease progression. **A,** Polar plots of DRGs (LRT interaction FDR<0.05) at 7 and 14 months. Radius represents log2 fold change in amplitude (log2 amp APP23 – log2 amp NTG) and angle represents phase difference (θ APP23 – θ NTG, hrs). **B,** mRNA expression and harmonic fits for four differentially rhythmic core clock genes at 7-months across cortical layers (error bars show mean±SD, * p<0.1, ** p<0.05). **C,** Polar plots of phase and log2 amplitude in NTG and APP23-TG mice. Arrows represent mean resultant vectors across cortical layers with vectors colored by genotype. **D,** Spatial map of *Csrnp1* phase difference between APP23-TG and NTG mice projected onto the Allen Brain Atlas at 7 and 14 months. **E,** mRNA expression and harmonic fits for *Csrnp1* in cortex layer 2/3 at 7 and 14 months (error bars show mean±SD, * p<0.1, ** p<0.05). **F,** Same as E for the gene *Egr1* at 7 months**. G**, Phase for genes with significant genotype:rhythm interaction in a full joint model including both ages (LRT FDR<0.1). **H,** Transcription‐factor co‐regulatory network for the gene set shown in G. Nodes represent TFs whose binding motifs are enriched in DRG promoters (ChEA3); edge thickness reflects the strength of predicted co‐regulation. DRGs (FDR<0.05) are highlighted red. **I,** Bubble plot of KEGG pathway enrichment of DRGs (APP23-TG vs. NTG).

Differential rhythmicity testing revealed widespread shifts in the phase and/or amplitude of daily gene expression rhythms in APP23-TG mice relative to NTG controls at 7 months of age (Fig. 6A, Supplementary Table S10). These shifts were especially pronounced in cortex and in the subgranular zone of the dentate gyrus of the hippocampus, and included both phase advancement and delay. While some genes were only modestly shifted, others had opposite phase (12 h shift) in APP23-TG mice, such as *Nr4a1*, *Dusp1*, and *Trib1*. Notably, core clock genes *Per1* and *Per2*, *Cry2*, and *Bhlhe40* (DEC1) were phase-delayed by ~6 h in APP23-TG mice compared to NTG (Fig. 6B,C). Consistent with this shift in the core molecular clock, a cortex-wide phase map revealed that many DRGs in cortical clusters had similar phase delays. By contrast, phase advances were more variable across cortical layers (Fig. 6G).

The activity-induced transcription factor, *Egr1,* had significantly stronger rhythmicity in APP23-TG compared to NTG at 7 months (Fig. 6F; interaction LRT FDR<0.05) but returned to very low amplitude oscillation in APP23-TG mice at 14 months. Another transcriptional regulator, *Csrnp1* (cysteine-serine-rich nuclear protein 1), stood out for its pronounced phase delay (10 h). *Csrnp1,* which has been linked to genetic risk for AD(57), was significantly phase-delayed in cortex layer 2/3 of APP23-TG mice at 7 and at 14 months of age (Fig. 6E; interaction LRT FDR<0.05).

To identify potential drivers of these expression phase shifts, we performed transcription factor motif enrichment analysis on the DRGs using ChEA3(58). We found significant enrichment of binding motifs in DRG promoters, including transcription factors *Csrnp1*, *Jun*, *Atf3*, *Fos*, and *Egr1* (Fig. 6H, FDR<0.05, Supplementary Table S11). Interestingly, many of these factors are immediate-early genes that are transcriptionally induced by neuronal activity.

By 14 months, and coinciding with increased amyloid burden, most cortical and hippocampal transcript that gained rhythmicity at 7 months were no longer differentially expressed (Fig. 6A). This finding supports that pre-amyloid changes in rhythmicity may be an adaptive response to early pathological changes, which is later overridden by neurodegeneration as disease advances.

Despite their relative scarcity, DRGs detected at 14 months in APP23-TG mice were functional for pathways directly linked to pathology, including neurodegeneration and cellular stress (FDR<0.05, Fig. 6I, KEGG analysis). Top enriched pathways specifically included Alzheimer’s disease, MAPK signaling, autophagy, and transcriptional misregulation, indicating that DRGs converge on key disease-relevant processes (e.g., protein homeostasis, metabolism, and stress signaling). Together, these findings demonstrate that APP23-TG mice have pronounced, region-specific perturbations in diurnal gene expression timing characterized by phase and amplitude changes in the core molecular clock and relayed to first and second-order output genes that integrate networks fundamental to neurodegeneration.

## Discussion

This study presents a brain-wide, Zeitgeber time (ZT)-resolved spatial transcriptomic atlas of the mouse brain that maps the landscape of brain region-specific diurnal gene expression in health and along the progression of Alzheimer’s pathology. The regulation of 24-hour rhythmicity in the brain is critical for healthy cognition (2, 59–62). Whole-brain microscopy has shown that circadian rhythms are ubiquitous across brain regions(2). Transcriptome sequencing further characterized the extent of rhythmic gene expression in cortex and hippocampus(9), and single cell RNA-seq of the whole brain confirmed cell type specific regulation of light- vs. dark-phase gene expression(7). Our study now provides a spatially resolved, whole-transcriptome map of diurnal gene expression as a foundation for understanding the region-specific regulation of circadian rhythms in health and disease.

Across the brain, our data show that the phase of core-clock gene rhythms are aligned, but the number and composition of rhythmic transcripts differ by region. Subfields of the hippocampal formation and upper cortical layers have the largest number of rhythmic genes(9), while subcortical regions and white matter tracts had lower levels of rhythmicity. The phase of peak expression has a bimodal distribution, with most genes peaking several hours before the light/darkness transitions. A notable exception was the thalamic reticular nucleus, a key regulator of sleep(30)(31), in which several dozen rhythmic genes peak around ZT4. This finding highlights the importance of spatially resolved rhythmic gene expression analysis for a deeper understanding of brain function.

Our ST data reveal differences in rhythmic gene expression along major axes of the cortex and hippocampus. Notably, we found a profound difference in rhythmic gene expression between the caudal visual cortex and rostral sensorimotor areas. Rhythmic expression of core clock components such as *Dbp* was attenuated in caudal cortex, while other genes like *Elk1* had stronger rhythmicity in caudal compared with rostral cortex. A group of neural activity-regulated genes, including *Egr1* and *Egr3*, presented antiphasic expression in caudal vs. rostral cortex. These results are consistent with a light-driven increase of neural activity in visual cortical areas, whereas motor and somatosensory areas are mainly activated during the dark phase, coinciding with the active phase in mice. In the hippocampus, we confirmed previous reports of large differences in mesor, or mean expression, for hundreds of genes between the dorsal and ventral subfields (32, 33, 35). Despite these differences in the mesor, diurnal rhythms were largely preserved across the dorsal-ventral axis and we found few differentially rhythmic genes.

Our comprehensive ST data allowed us to analyze alterations in rhythmic transcription in the APP23 model of AD, showing that the brain regions most vulnerable to Aß pathology presented the greatest alterations in the rhythmic transcriptome. Notably, at 7 months of age, before widespread plaque deposition, we observe brain-wide expansion of rhythmic genes and an increase in rhythmic amplitude, especially in cortex. These data extend observations of dysregulated rhythmic gene expression in other models of AD pathology, providing novel spatial context(9, 48, 63, 64). In our study, core clock transcripts show phase delays of several hours at 7 months, indicating a shift in molecular timing that parallels circadian behavioral disturbances. The observation of dramatic changes at early disease stages highlight the potential role of circadian dysregulation as a driver or amplifier of subsequent pathological cascades.

Genes that gained rhythmicity in APP23-TG are enriched for activity-regulated immediate-early genes, suggesting neuronal activation may have greater temporal structure in APP23-TG. In cortex, an activity-based gene score was more rhythmic in APP23-TG, supporting this interpretation. Among the IEGs, *Csrnp1* had a robust rhythm with a marked phase delay at seven months that remains detectable, though attenuated, at fourteen months, suggesting that some timing changes persist as disease advances. Importantly, our data do not distinguish gene expression changes which may cause circadian disruption from those which are consequences of altered rhythmicity. Future experiments could test the causal roles of genes highlighted here.

Limitations qualify these conclusions. Our ST data have spatial resolution of ~50 μm, and each spot includes transcripts from 1–5 or more cells. This can obscure information, for example about genes which oscillate with different phases in different cell types. Single cell sequencing can help to resolve such differences, but previous studies have included a limited number of ZT samples (e.g. 2 time points(7)). We designed our study with four ZT samples to allow estimation of both phase and amplitude for diurnal gene expression rhythms across the brain. We focused on a single model of amyloid pathology, APP23-TG, and two ages; extending these studies to tauopathy models, additional ages, and/or human tissue will establish the general validity of our findings. Our data set the stage for these investigations and establish the critical importance of accounting for diurnal rhythmicity in analyses of cell- and region-specific impacts of neurodegeneration.

## Supplementary Material

Supplement 1

## Data Availability

The raw and processed spatial transcriptomics data from this study are available at NCBI accession GSE282203.
