# Supplementary material for: Spatial transcriptomics reveals brain-wide circadian disruption in an Alzheimer’s disease model
Source: bioRxiv. 2026 Jan 28:2026.01.26.701799. Preprint. [Version 1] doi: 10.64898/2026.01.26.701799 (PMC12874051; doi:10.64898/2026.01.26.701799)
Supplement: Supplement 1 [file NIHPP2026.01.26.701799v1-supplement-1.pdf]

bioRxiv preprint doi: https://doi.org/10.64898/2026.01.26.701799; this version posted January 28, 2026. The copyright holder for this preprint (which was not certified by peer review) is the author/funder, who has granted bioRxiv a license to display the preprint in perpetuity. It is made available under aCC-BY 4.0 International license.

Materials and Methods

Experimental model details

APP23 transgenic (APP23-TG) mice (B6.Cg-Tg(Thy1-APP)3Somm/J; RRID:IMSR_JAX:030504) and non-transgenic (NTG) littermates control mice were housed in light-tight enclosures at the University of California, San Diego. The mice were given ad libitum food (Teklad rodent diet 8604; Envigo, Indianapolis, IN) and water access. This study used a total of 65 APP23 mice almost equally distributed across sex and genotype. The work presented in this study followed all guidelines and regulations of the UCSD Division of Animal Medicine that are consistent with the Animal Welfare Policy Statements and the recommendations of the Panel on Euthanasia of the American Veterinary Medical Association.

Experimental design

Prior to beginning any experiments, mice were habituated to a 12h:12h light-dark (LD 12:12) cycle and single housing conditions in custom light-tight cabinets. We then measured diurnal rhythms in locomotor activity (see below). By definition, Zeitgeber time 0 (ZT0) is the time when lights go on and ZT12 is the time when the lights go off. After behavior tests were completed, mice within each genotype and treatment group were randomly assigned for sacrifice at one of four time points: ZT0, ZT6, ZT12, or ZT18. ZT0 and ZT6 tissues were collected in the light; ZT12 and ZT18 were collected in the dark.

Monitoring of cage locomotor activity

Experimental mice were singly housed in standard cages equipped with IR motion sensors (Actimetrics) and locomotor activity data were acquired using ClockLab Wireless Data Collection and analyzed using ClockLab Analysis 6 (Actimetrics) as previously described(*9*). Mice were entrained to a 12:12 LD cycle. Activity data in LD were collected for at least two weeks. The amount of cage activity (number of IR beam breaks) over a 24 h period was averaged over the recording period and reported in arbitrary units (a.u.)/h. The average number of activity bouts and the average bout lengths were determined for 24 h and for light and dark periods, with new activity bouts defined following an inactivity gap of either 21 min (maximum gap: 21 min; threshold: 3 counts/min) or 1 min (maximum gap: 1 min; threshold: 3 counts/min).

To determine free running period, an independent cohort of mice were placed into constant darkness (DD) for two weeks following LD activity recording. These mice were not used for spatial transcriptomics. Free running period was obtained from the slope of a line fitted to the daily activity onset times during the DD period.

bioRxiv preprint doi: https://doi.org/10.64898/2026.01.26.701799; this version posted January 28, 2026. The copyright holder for this preprint (which was not certified by peer review) is the author/funder, who has granted bioRxiv a license to display the preprint in perpetuity. It is made available under aCC-BY 4.0 International license.

Tissue collection

Mice were euthanized with CO_2 followed by decapitation, either in the dark (ZT12 and ZT18) or in the light (ZT0 and ZT6). Brain hemispheres were collected and placed in OCT and then flash frozen in isopentane in liquid nitrogen. One hemibrain from each mouse was cryosectioned at -18°C sagittally to a thickness of 10 µm (2.8 mm from the midline) using a standard Leica CM1860 cryostat and processed according to the recommended protocols (Tissue optimization: CG000240 Visium 10X Genomics; Gene expression: CG000239). The tissue was immediately mounted on a Visium spatially barcoded slide (10X genomics). The remaining tissue was covered with OCT and kept at -80 °C until it was cryosectioned again starting at the same position to a thickness of 10 µm and mounted onto a Superfrost plus microscope slide (Fisherbrand) for staining. Each section covered approximately 80% of the 5,000 total spots within their fiducial frame. Slides were stored at -80 °C until use.

Spatial transcriptomics (ST)

Visium spatial gene expression slides and reagents were used according to the manufacturer instructions (10X Genomics). Each capture area was 6.5mm x 6.5 mm and contained 5,000 barcoded spots that were 55 µm in diameter (100 µm center to center between spots), providing an average resolution of about 1 to 10 cells per spot. Optimal permeabilization time was measured at 24 min. Libraries were prepared according to the Visium protocol (10X genomics) and sequenced on a NovaSeq4 (Illumina) at a sequencing depth of 182 million read-pairs by the UCSD genomics gore (IGM). Sequencing was performed with the recommended protocol (read 1: 28 cycles; i7 index read: 10 cycles; i5 index read: 10 cycles; and read 2: 100 cycles), yielding between 175.8 million and 187.9 million sequenced reads. H&E (Hematoxylin, Thermo Cat. No. Thermo; Dako bluing buffer, Dako Cat. No. CS702; Eosin Y, Sigma, Cat. No. 1.09844.1000) staining and image preparation was performed according to the Visium protocol. H&E-stained sections were imaged using a Nanozoomer slide scanner (Hamamatsu) Spatial gene expression assay was performed according to the protocol CG000239. Samples with a sequencing saturation below 50% were sequenced again.

Pre-processing libraries

Each Visium slice was processed using the SpaceRanger software provided by 10X genomics for mapping reads, aligning spot barcodes to the brightfield tissue image, and generating UMI count table tables. The human APP23 transgene was added to mm10 2020-A reference in order to be able to later quantify transgene expression. As a preliminary quality control measure we analyzed each slice individually using the Seurat ST module(*65*) and inspected the distribution of number of genes detected, number of UMIs, percent mitochondrial reads and number of barcodes passing UMI thresholds. We excluded 4 slices from downstream analysis that had median detected features per spot of less than 500 genes with over 90% sequencing saturation.. Lastly we verified that male and female samples expressed the appropriate sex markers which

bioRxiv preprint doi: https://doi.org/10.64898/2026.01.26.701799; this version posted January 28, 2026. The copyright holder for this preprint (which was not certified by peer review) is the author/funder, who has granted bioRxiv a license to display the preprint in perpetuity. It is made available under aCC-BY 4.0 International license.

led us to exclude one more slice due to coexpression of Xist and Y chromosome genes. This yielded 65 high quality, with each combination of experimental covariates having at least 2 replicates except 14 month, non-transgenic males at ZT0 which only had one replicate.

Integrated Clustering

We used the method PRECAST and performed parameter exploration to find a clustering that best corresponded with the Allen Brain Mouse Reference Atlas [10] both anatomically and transcriptomically. PRECAST allows for integrated clustering across multiple ST datasets while enforcing spatial smoothness and accounting for complex batch effects by computing latent factors with spatially informed priors. Our final clusters were generated by first filtering out spots with less than 500 genes detected and using the following PRECAST parameters: 25 candidate clusters, 25 latent factors, q=25, and 3000 spatially variable genes detected by the SPARK-X package. This yielded 25 final clusters, 1 of which was judged to be of low quality due to a lower mean number of genes detected than other clusters and was removed. 2 clusters corresponded to fiber tracts and spanned anatomical regions such as corpus callosum and medial forebrain bundle system and were merged into one cluster annotated as fiber tracts. This resulted in 23 final anatomically annotated clusters. Marker genes were identified using Seurat's FindMarkers function and cluster markers were verified to correspond to their anatomical annotation using the Allen Brain Atlas *in situ* hybridization atlas.

Pseudobulk generation

As a first step to perform cluster-specific rhythmicity analyses we generated pseudobulk gene expression profiles for each cluster and excluded genes that had less than 10 counts in at least one sample. This yielded roughly 12,000 expressed genes per pseudobulk profile. We also excluded samples from any cluster pseudobulk profile that had less than 20,000 total counts or expressed less than 90% of the genes following count thresholding because this indicated the cluster was underrepresented in the sample. After QC 21 of the 23 clusters included at least 28 of the 33 samples. In particular, across all cortical and hippocampal clusters, at least 32 of 33 samples passed these QC thresholds.

Immunohistochemistry

For immunohistochemistry (IHC), separate age-matched mice were sacrificed and both brain hemispheres were extracted. One hemi-brain was fixed by 4% paraformaldehyde. Fixed APP23 brains were sectioned sagitally at 40 µm using a Leica VT1000S vibratome. Sections were washed three times in PBS, pre-treated with 1% Triton X-100, 10% H_2O_2 in PBS for 20 min at room temperature, washed again, and incubated for 1 h at room temperature in 10% serum according to secondary antibody species. The sections were incubated with primary antibodies to microglia marker Iba1 (1:500, FUJIFILM Wako Chemicals U.S.A. Corporation, code number

bioRxiv preprint doi: https://doi.org/10.64898/2026.01.26.701799; this version posted January 28, 2026. The copyright holder for this preprint (which was not certified by peer review) is the author/funder, who has granted bioRxiv a license to display the preprint in perpetuity. It is made available under aCC-BY 4.0 International license.

019-19741), neuronal marker NeuN (1:200, MiliporeMillipore Corp., Cat. No. MAB377), amyloid marker 82E1 (1:500, Immuno-biological Laboratories, Cat. No. 10323), and astrocyte marker GFAP (1:200, Invitrogen, Cat. No. PA5-16291) at 4°C overnight. Sections were washed three times, incubated in 1:100 biotinylated secondary antibody (goat anti-rabbit, Vector Laboratories, Cat. No. BA-1000; or horse anti-mouse Vector Laboratories, Cat. No. BA-2000) for 30 min at room temperature, washed again, incubated in biotinylated HRP and avidin (ABC, Vector Laboratories, Cat. No. 30015 and 30016PK-6100) for 1 h in the dark at room temperature and then treated with diaminobenzidine (DAB) Substrate Kit, Peroxidase (Vector Laboratories, Cat. No. SK-4100) for coloration. 20X images were collected using an Olympus VS200 Slide Scanner and analyzed using ImageJ. Cortex GFAP+ area values were normalized to the total cortex area for each mouse.(*9*)

Plaque analysis

To identify genes that are differentially expressed in plaque-associated spots versus normal spots in the cortex, we used a generalized linear mixed-effects model. Specifically, we labeled all spots as either plaque associated or non-plaque associated, and predicted gene expression with plaque association as a fixed effect and sample as a random effect, to account for cross-sample variability. We performed a likelihood ratio test comparing models with and without plaque fixed effects, to assess significance of coefficients. To estimate the effect of plaque distance on gene expression we calculated the distance of each spot to the nearest plaque-associated spot. Only spots with a distance to nearest plaque spot less than 600um were included for analysis. We used another mixed effects models to model gene expression as a function of plaque distance, with a random intercept for sample as above. We performed a likelihood ratio test comparing models with and without plaque distance as a fixed effect. Finally, we modeled the relationship between DAM score and plaque distance, with a random intercept for sample. Here, we used a Wald's test to test for significance of coefficients. For all analyses, plaque FDR correction was used with a cutoff of 0.1.

Supplementary Text

Rhythmicity detection and differential rhythmicity analysis

Next we develop a method based on negative binomial regression which tests for rhythmicity while controlling for sex and age (. We model the expression of each gene with a rhythmic component expressed as a sinusoidal function of Zeitgeber time (ZT). This function can be expressed as a linear combination of two Fourier components, which is therefore compatible with a generalized linear model (GLM) framework. To illustrate this let y_t represent normalized counts of a gene at ZT=t, let A represent the amplitude, ϕ represent the phase, and y_0 represent the mesor. Then our model is as follows:

bioRxiv preprint doi: https://doi.org/10.64898/2026.01.26.701799; this version posted January 28, 2026. The copyright holder for this preprint (which was not certified by peer review) is the author/funder, who has granted bioRxiv a license to display the preprint in perpetuity. It is made available under aCC-BY 4.0 International license.

$$y_t = y_0 + A \cdot sin(\frac{2\pi}{24}t + \phi)$$
$$y_t = y_0 + A \cdot (cos(\phi) \cdot sin(\frac{2\pi}{24}t) + sin(\phi) \cdot cos(\frac{2\pi}{24}t))$$
$$y_t = y_0 + a \cdot sin(\frac{2\pi}{24}t) + b \cdot cos(\frac{2\pi}{24}t)$$

Where a and b represent unknown linear parameter to be fit:

$a = A \cdot cos(\phi)$, $b = A \cdot sin(\phi)$

The harmonic model can be tested for significance by removing the ***a*** and ***b*** coefficients and running a likelihood ratio test with the full and reduced model. Once the model is fit estimate amplitude, phase can be recovered via:

$A = \sqrt{a^2 + b^2}$, $\phi = tan^{-1}(\frac{b}{a})$

This approach has the benefit of allowing both for the testing of interaction terms directly and fitting age, sex and genotype-specific amplitude and phase all within this same model, unlike standard tools of detecting rhythmicity such as JTK_CYCLE and ARSER. While the approach does not allow for the estimation of period, our experimental approach with four ZT points precludes this possibility. Thus we limit our scope to genes with 24-hour periodicity.

We examined rhythmicity detection results from the glm method described above and found that they were largely consistent with previously established methods implemented in the R package MetaCycle. For genes called as rhythmic by either MetaCycle and/or our approach, there is a high degree of correlation for phase estimates, p-values and relative amplitude estimates. In addition, there is a high degree of overlap in the genes passing chosen FDR thresholds by both methods.

To identify differentially rhythmic genes, we first defined sets of rhythmic genes for each cluster by the method described above in NTG animals and APP23-TG animals separately. For each cluster we then took the union of genes identified in the two genotypes as candidates for genotype-rhythmicity interaction testing. We then ran a likelihood ratio test which included a term modeling the interaction between genotype and the harmonic coefficients and tested it against a reduced model lacking this term. I.e

Full: $\sim age + sex + genotype + a + b + age:(a + b) + sex:(a + b) + genotype:(a + b)$

Reduced:

bioRxiv preprint doi: https://doi.org/10.64898/2026.01.26.701799; this version posted January 28, 2026. The copyright holder for this preprint (which was not certified by peer review) is the author/funder, who has granted bioRxiv a license to display the preprint in perpetuity. It is made available under aCC-BY 4.0 International license.

$$\sim age + sex + genotype + a + b + age:(a + b) + sex:(a + b)$$

Permutation-based analysis to control false discovery rate

We used an empirical, permutation-based procedure to ensure that our statistical analysis of differentially rhythmic genes had the expected level of control for false discoveries. We created an empirical null distribution by randomly shuffling the genotype labels of each sample and computing the mean difference in relative amplitude of rhythmic genes. We repeated this shuffling procedure 500 times, and calculated empirical p-values as the fraction of permutations with mean difference greater than that observed in the real data. P-values were adjusted for multiple comparisons across clusters (Fig. 5C).

Phase density estimation

We took genes identified as rhythmic at an FDR threshold of 0.05 and used the phase estimates to compute a phase density estimation. In brief, kernel density estimation was performed using a Von Mises kernel, and the resulting density estimations were normalized first in the linear sense (by dividing the density estimate by the sum over all density estimates and the bin width, enforcing a unit area under the estimated density curve) and then for visualization purposes radar plot normalization was performed to normalize the integral of the square of densities in the phase space to 1 (*29*).

Differential Expression

In order to characterize differential gene expression between APP23-TG and NTG mice across brain regions, we used the same pseudobulk approach and quality control thresholds as input to DESeq2(*25*). For this analysis we reverted to using the Wald test modules of DESeq, as a likelihood ratio test was unnecessary without modeling harmonic effects and it allowed us to compare various combinations of covariates within the same model. After QC thresholding, we tested the effects of genotype, genotype:sex interactions, and genotype:age interactions and visualized the results.

DAM Score

To understand microglial activation in response to plaque accumulation we devised an approach to compute a disease associated microglia (DAM) score for each cluster in each genotype-age combination. The score is calculated by first taking a list of genes reported to be involved in microglial activation in AD, and subsetted the list to those that were found to be expressed in our

bioRxiv preprint doi: https://doi.org/10.64898/2026.01.26.701799; this version posted January 28, 2026. The copyright holder for this preprint (which was not certified by peer review) is the author/funder, who has granted bioRxiv a license to display the preprint in perpetuity. It is made available under aCC-BY 4.0 International license.

data. We then computed the principal components for each cluster-genotype-age combination using the log(CPM+1) values only for those genes. We then plotted the mean score for PC1 for each cluster in spatial coordinates and ran t-tests on the mean DAM scores per cluster and sample between genotypes and ages.

10μm-thick sections that were adjacent to the those used for ST were mounted onto Superfrost plus microscope slides (Fisherbrand) and fixed with 100% methanol for 30 minutes at -20°C. At room temperature, slides were briefly air dried and stained with 0.05% Thioflavin S (Sigma, Cat. No. T1892) in 50% EtOH for 8 minutes followed by three 5-minute washes with 80% EtOH. Sudan black B (0.1% in 70% EtOH) was then applied for 10 minutes, followed by three 5 minute washes with 80% EtOH, one 5 minute was with PBS, and 5 minute was with water. Sections were then coverslipped with Invitrogen ProLong™ Gold Antifade Mounting Medium with DAPI and imaged using an Olympus VS200 Slide Scanner. Due to the difficultly in sectioning the adjacent slide at the same angle after having removed it from the sectioning block, several of the stained sections were cut off at one end. To capture the adjacent tissue across the entire section, multiple 10 μm sections were stained and overlaid. To determine which spots in the Visium slide used for ST were adjacent to Ab plaques, the Thioflavin S-stained sections were overlaid onto the original Visium H&E-stained image. Spots containing or directly in contact with a plaque are defined as plaque associated spots.

<u>Neural activity score rhythmicity analysis</u>

To estimate neural activity scores, we applied the NeuroEstimator package to pseudobulk profiles in APP23-TG and NTG mice at 7 and 14 months. Neural activity scores were calculated separately for each sample and brain region using default parameters for the package.

To test for rhythmicity, we fit a harmonic regression model, modeling neural activity scores as a sinusoidal function of Zeitgeber time (ZT). A likelihood ratio test (LRT) was performed to compare models with and without harmonic terms. False discovery rate (FDR) correction was applied with a threshold of $q < 0.05$ to identify significantly rhythmic clusters. Analyses were performed independently for each genotype and age group.

<u>Pathway enrichment analysis</u>

Pathway enrichment analyses were performed to interpret differentially expressed genes (DEGs) and differentially rhythmic genes (DRGs). Depending on the analysis context, enrichment testing was conducted using either Gene Set Enrichment Analysis (GSEA) or Over Representation Analysis (ORA). GSEA was performed using the WebGestaltR package(*46*) with the KEGG pathway database. ORA was performed using the enrichKEGG function from the clusterProfiler R package(*66*).

bioRxiv preprint doi: https://doi.org/10.64898/2026.01.26.701799; this version posted January 28, 2026. The copyright holder for this preprint (which was not certified by peer review) is the author/funder, who has granted bioRxiv a license to display the preprint in perpetuity. It is made available under aCC-BY 4.0 International license.

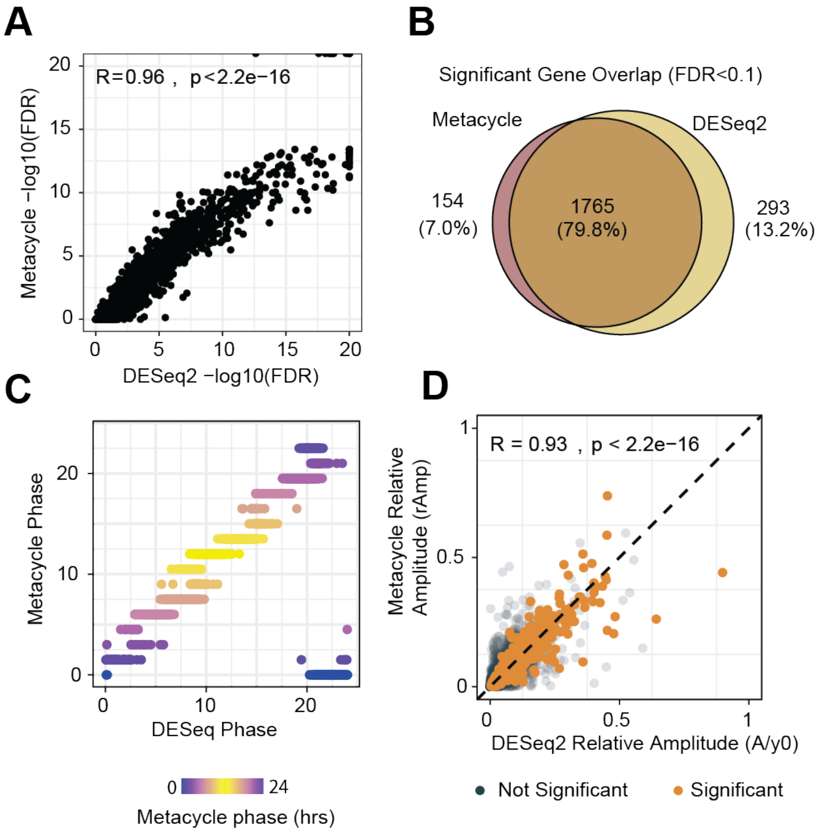

Figure S1. Validation of harmonic regression rhythmicity detection with DESeq2 in cortex layer 2/3 non-transgenic samples A, Scatterplot of -log10(FDR) values called by MetaCycle versus DESeq2 harmonic regression (spearman correlation=0.96). **B,** Venn diagram of genes passing rhythmicity FDR<0.1 by each method. **C,** Comparison of phase estimates (ZT of peak expression, in hours) from DESeq2 versus MetaCycle phase for significantly rhythmic genes. **D,** Scatter plot of relative amplitude estimates from DESeq2 versus MetaCycle's meta2d relative amplitude with significantly rhythmic genes (FDR<0.1) highlighted in orange (Spearman correlation=0.93, p<1e-15).

bioRxiv preprint doi: https://doi.org/10.64898/2026.01.26.701799; this version posted January 28, 2026. The copyright holder for this preprint (which was not certified by peer review) is the author/funder, who has granted bioRxiv a license to display the preprint in perpetuity. It is made available under aCC-BY 4.0 International license.

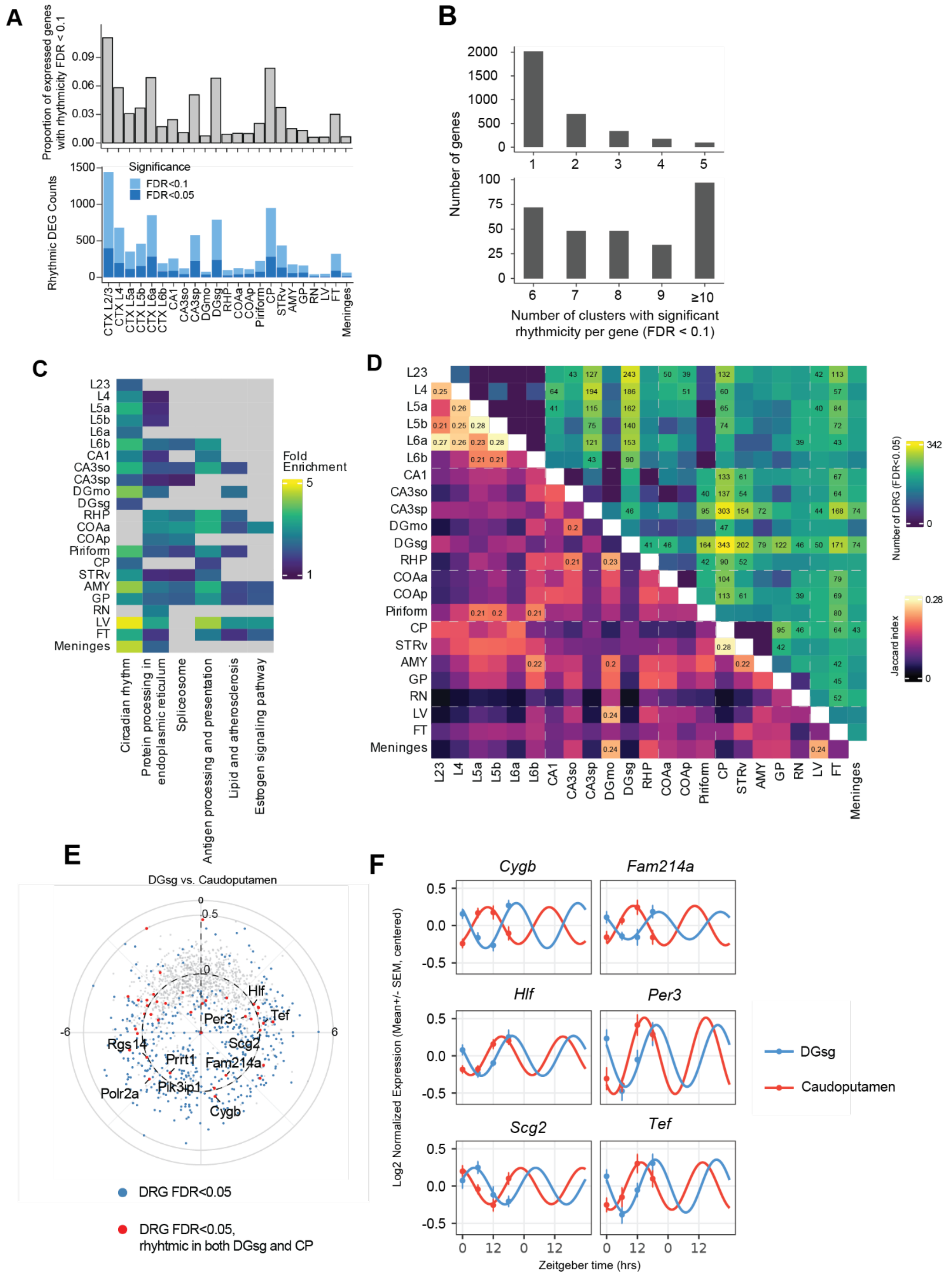

Figure S2. Comparative analysis of gene expression rhythms across brain regions in nontransgenic animals
A, Top, proportion of expressed genes in each cluster with rhythmicity FDR < 0.1. Bottom, number of rhythmic genes, separated by significance threshold (light blue, FDR < 0.1; dark blue, FDR < 0.05). **B,** Number of genes that are significantly rhythmic (FDR < 0.1) in a given number of clusters. The top panel summarizes genes rhythmic in 1–5 clusters, and the bottom panel summarizes genes rhythmic in 6–9 or ≥10 clusters. **C,** Enrichment of KEGG pathways for

bioRxiv preprint doi: https://doi.org/10.64898/2026.01.26.701799; this version posted January 28, 2026. The copyright holder for this preprint (which was not certified by peer review) is the author/funder, who has granted bioRxiv a license to display the preprint in perpetuity. It is made available under aCC-BY 4.0 International license.

rhythmic genes (FDR<0.05). Terms found in >1 cluster are shown. Grey cells indicate no significant enrichment (FDR ≥ 0.05). **D,** Overlap of rhythmic genes (bottom left) and number of DRGs (top right, LRT FDR<0.1) for all pairs of clusters. The cluster pairs with the largest overlap or Jaccard index are labeled with the numerical score. **E,** Polar plot of DRGs for an example comparison of two regions, dentate gyrus granule layer (DGsg) versus caudoputamen (CP), with highly distinct rhythmic gene sets. Selected genes with the lowest FDR and similar amplitude are labeled. **F,** mRNA expression and sin fits for DGsg (blue) and CP (red) for DRG. Points (± SEM) show centered log2-normalized expression at each Zeitgeber time, and lines show harmonic regression fits.

bioRxiv preprint doi: https://doi.org/10.64898/2026.01.26.701799; this version posted January 28, 2026. The copyright holder for this preprint (which was not certified by peer review) is the author/funder, who has granted bioRxiv a license to display the preprint in perpetuity. It is made available under aCC-BY 4.0 International license.

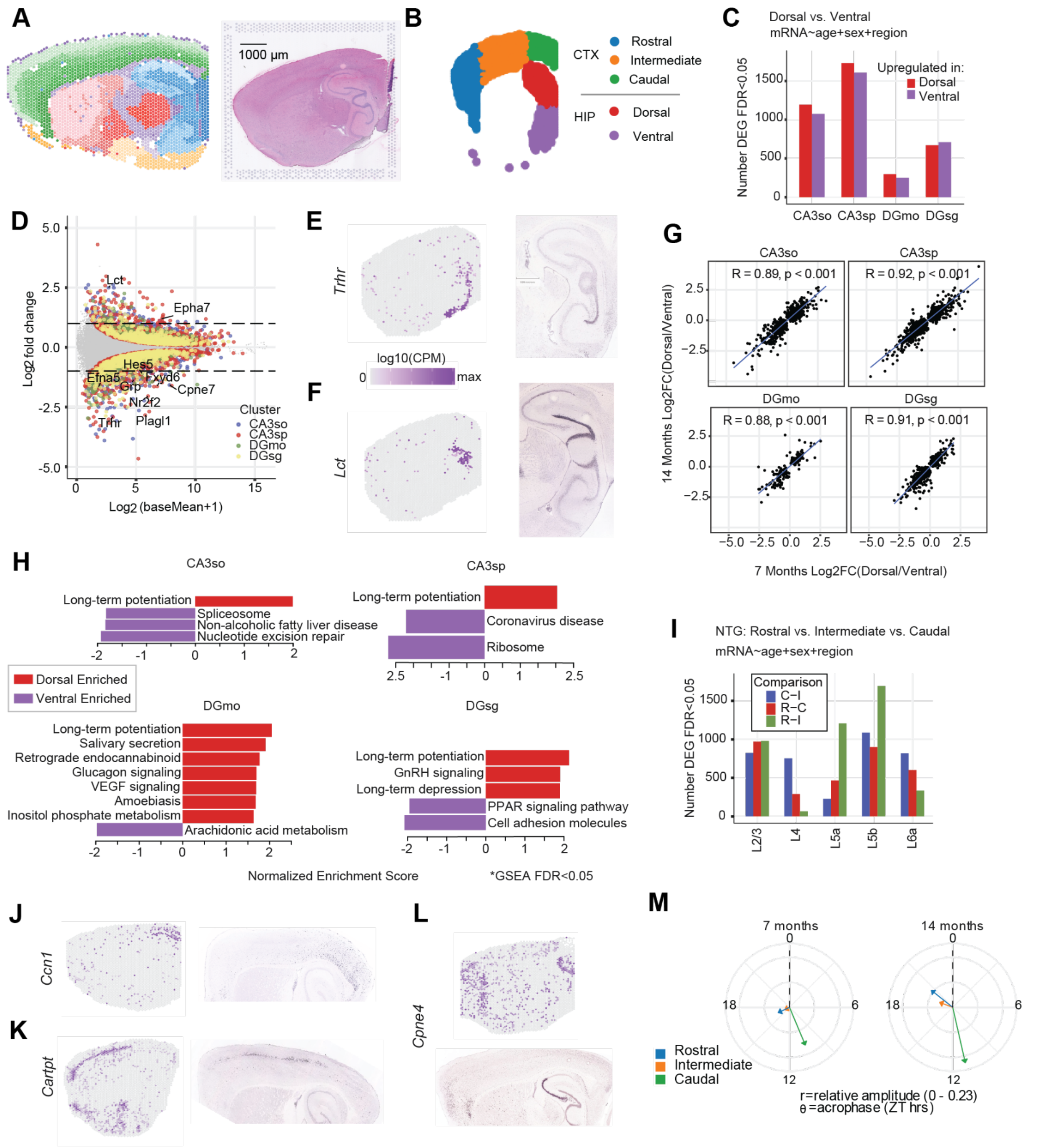

Figure S3. Dorsal versus ventral hippocampal transcriptional differences and relationship to cortical regionalization. A, Sagittal section from the spatial transcriptomic dataset showing clusters overlaid on the brain outline, capturing hippocampus and adjacent rostral, intermediate and caudal cortex. **B,** Annotation of dorsal and ventral hippocampus and rostral, intermediate and caudal cortex used for downstream analyses. **C,** Number of differentially expressed genes (DEGs) between dorsal and ventral hippocampus for each hippocampal cluster (mRNA ~ age + sex + region, FDR < 0.05). **D,** MA plot of dorsal versus ventral differential expression across hippocampal clusters **E,F,** Spatial expression of dorsal (*Trhr*) and ventral (*Lct*) marker genes in our dataset, and corresponding in situ hybridization images from the Allen Brain Atlas(*23*) (right). **G,** Concordance of dorsal versus

bioRxiv preprint doi: https://doi.org/10.64898/2026.01.26.701799; this version posted January 28, 2026. The copyright holder for this preprint (which was not certified by peer review) is the author/funder, who has granted bioRxiv a license to display the preprint in perpetuity. It is made available under aCC-BY 4.0 International license.

ventral log2 fold changes between 7-month and 14-month NTG mice for each hippocampal cluster. **H,** Gene set enrichment analysis of dorsal and ventral log fold changes (GSEA FDR < 0.05). **I,** Number of DEGs identified in pairwise comparisons between rostral, intermediate and caudal cortex across cortical layers (mRNA ~ age + sex + region, FDR<0.05). **J-L,** Spatial expression of representative cortical regional marker genes and matching Allen Brain Atlas in situ hybridization images. **M,** NeuroEstimator score rhythmic mean resultant vectors for rostral, intermediated and caudal cortex.

bioRxiv preprint doi: https://doi.org/10.64898/2026.01.26.701799; this version posted January 28, 2026. The copyright holder for this preprint (which was not certified by peer review) is the author/funder, who has granted bioRxiv a license to display the preprint in perpetuity. It is made available under aCC-BY 4.0 International license.

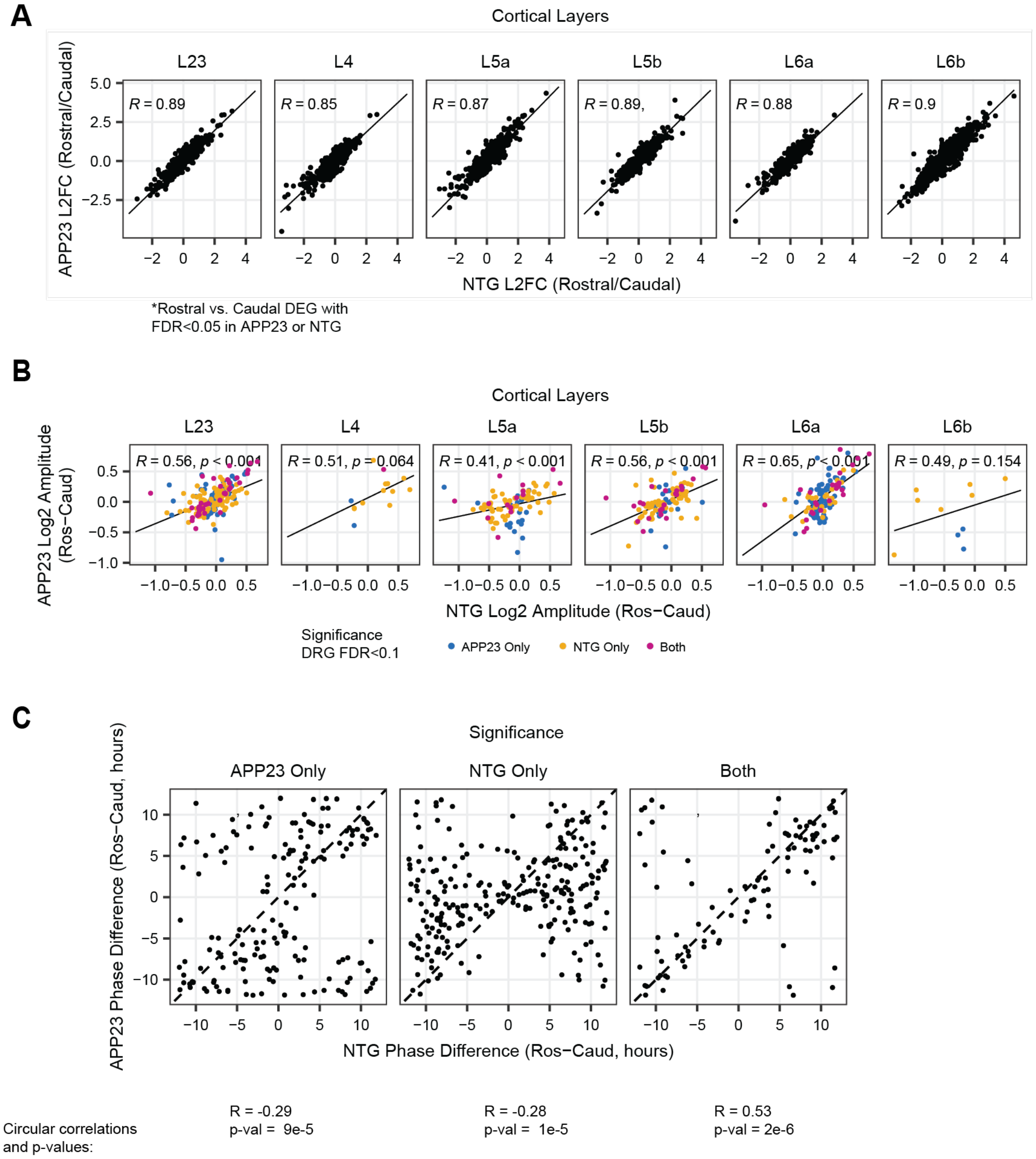

Figure S4: Comparison of differential expression and rhythmicity between rostral and caudal cortex in NTG and APP23 A, Scatterplots of rostral vs. caudal differential expression across cortical layers. Each point is a gene, plotted by its rostral/caudal log2 fold change in NTG (x axis) and APP23 (y axis) **B,** Scatterplots of rostral-caudal rhythmic amplitude differences in NTG (x axis) and APP23 (y axis) for differentially rhythmic genes (FDR< 0.1), colored by rostral-caudal differential rhythmicity significance in NTG only, APP23 only, or both **C,** For all cortical rostral-caudal DRGs (FDR<0.1) scatterplots of phase differences between rostral and caudal in NTG (x axis) and APP23 (y axis), faceted by rostral-caudal differential rhythmicity significance in NTG only, APP23 only, or both. Circular correlations and p values from the Circular R package are shown below(*67*)**.**

bioRxiv preprint doi: https://doi.org/10.64898/2026.01.26.701799; this version posted January 28, 2026. The copyright holder for this preprint (which was not certified by peer review) is the author/funder, who has granted bioRxiv a license to display the preprint in perpetuity. It is made available under aCC-BY 4.0 International license.

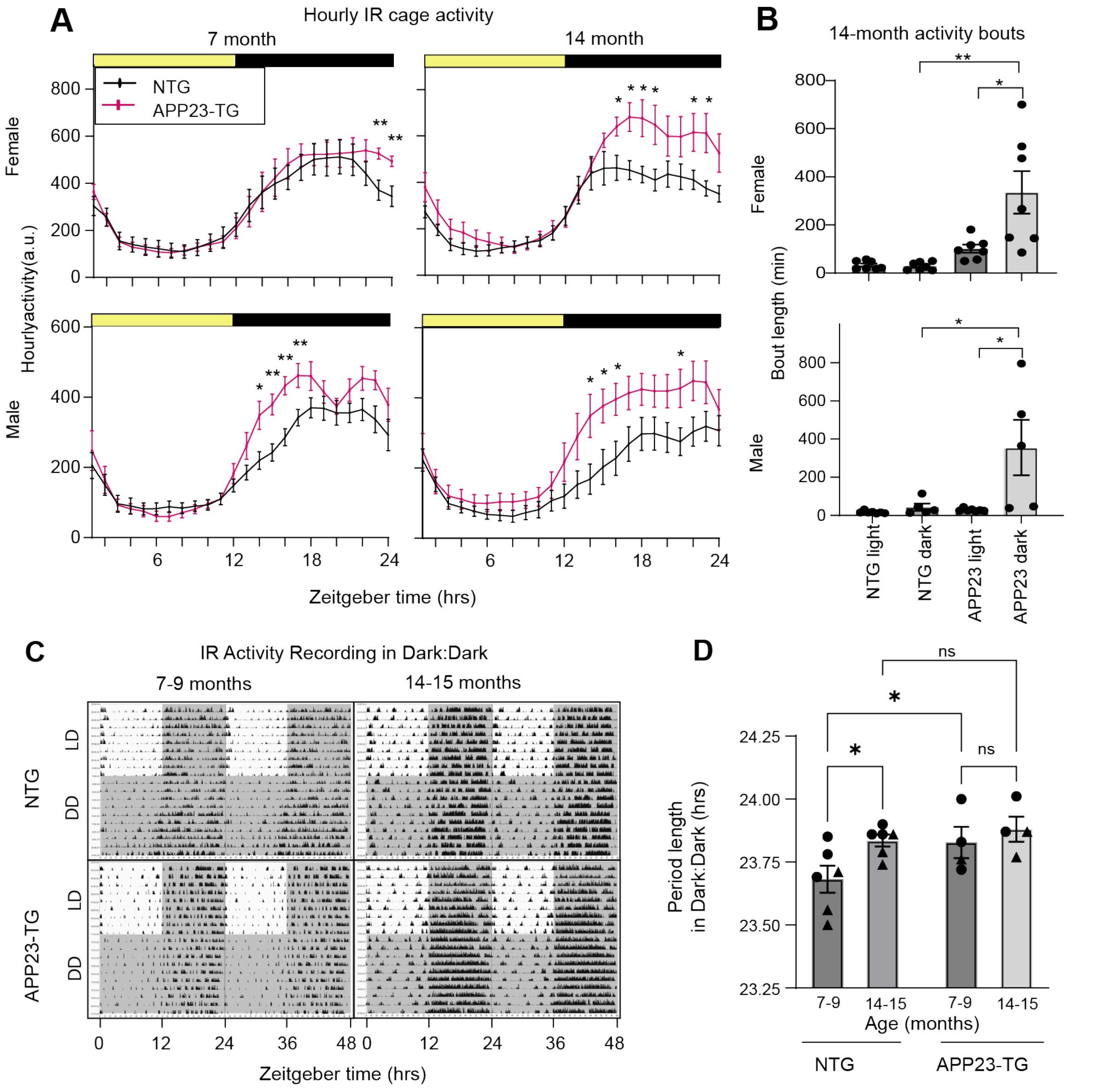

Figure S5: APP23-TG mice exhibit age-dependent alterations in circadian activity and period. A, Mean hourly infrared activity under a 12 h light–12 h dark (LD) cycle at 7 months and 14 months. Mean +/− SEM. Asterisks indicate significant genotype differences at individual Zeitgeber times (ANOVA: * $p \leq 0.05$, ** $p \leq 0.01$). **B,** Distribution of activity-bout lengths during the light and dark phases measured at 14 months. **C,** Representative actograms of home-cage activity under a 12:12 h light–dark cycle for 7- and 14-month NTG and APP23-TG mice, illustrating hyperactivity during the active period in APP23-TG animals. **D,** Free-running period measured in constant darkness following entertainment at 7 and 14 months of age. The free-running period was significantly longer in APP23-TG compared with NTG mice at 7 months of age (Two-tailed t-test, $p \leq 0.05$), but not at 14 months.

bioRxiv preprint doi: https://doi.org/10.64898/2026.01.26.701799; this version posted January 28, 2026. The copyright holder for this preprint (which was not certified by peer review) is the author/funder, who has granted bioRxiv a license to display the preprint in perpetuity. It is made available under aCC-BY 4.0 International license.

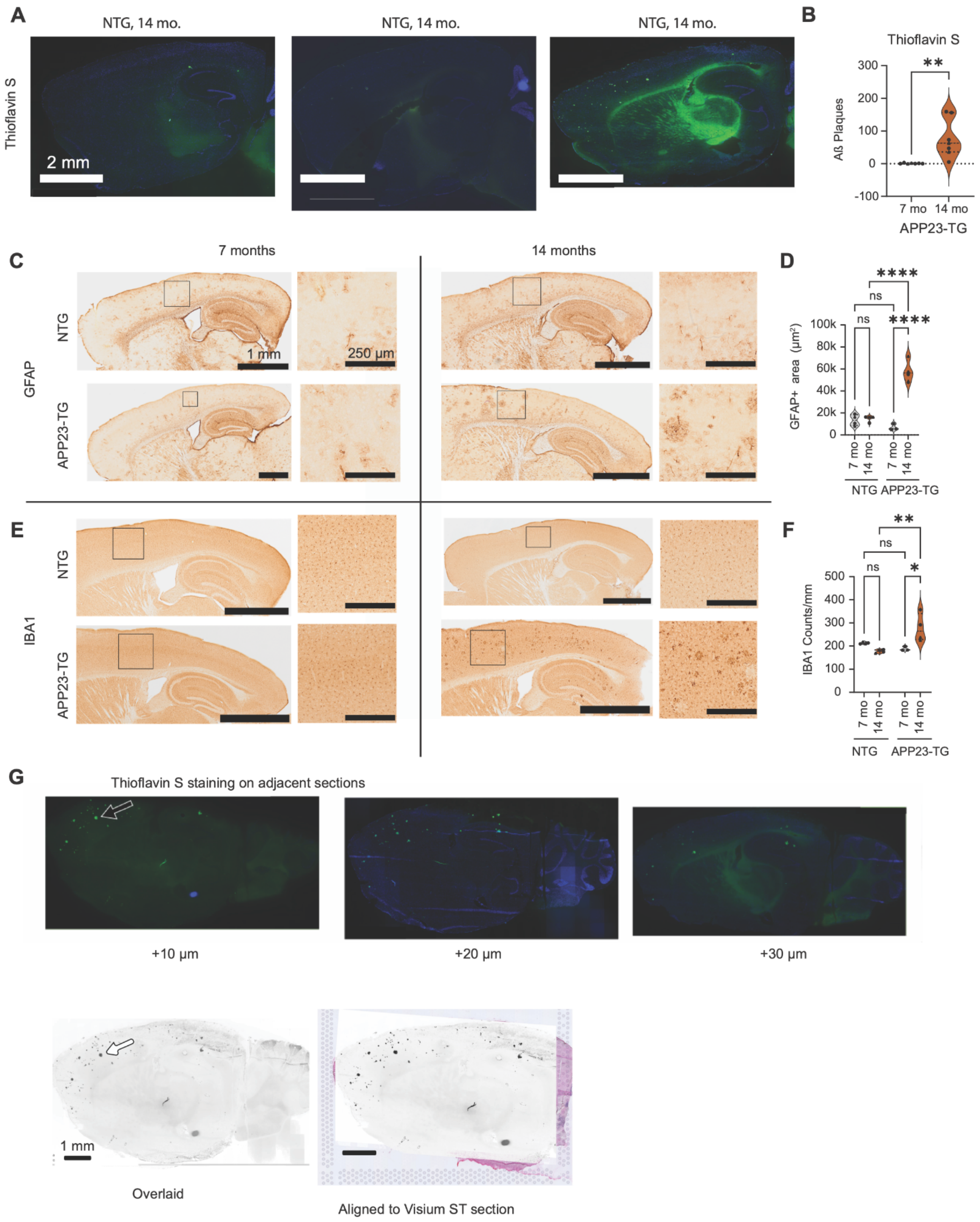

Figure S6: Progressive neuropathology in the APP23 mouse model of AD. A, Thioflavin S staining (green) of Aβ in 14-month old NTG and 7- and 14-month APP23-TG sections (scale bar = 2 mm; DAPI, blue). **B,** Cortical plaque counts in 7- and 14-month APP23-TG sections (n = 7; Two-tailed t test (unpaired); ** $p < 0.01$) (right). **C,** DAB staining for GFAP (astrocytes) 7- and 14-month NTG and APP23-TG sections. Low-magnification panels (scale bar = 1 mm) with outlines indicate cortical fields zoomed in (scale bar = 250 µm), illustrating progressive

bioRxiv preprint doi: https://doi.org/10.64898/2026.01.26.701799; this version posted January 28, 2026. The copyright holder for this preprint (which was not certified by peer review) is the author/funder, who has granted bioRxiv a license to display the preprint in perpetuity. It is made available under aCC-BY 4.0 International license.

astrogliosis. **D,** GFAP positive cortical area in 7- and 14-month NTG and APP23-TG sections (n = 4). **E,** DAB staining for IBA1, illustrating and emerging microgliosis. **F,** Cortical IBA1 counts in 7- and 14-month NTG and APP23-TG sections (n = 4) (bottom right). Circles represent females and triangles represent males (One-way ANOVA with Šídák's multiple comparison test; ns $p > 0.05$; * $p \leq 0.05$; ** $p \leq 0.01$; **** $p \leq 0.0001$). **G,** Thioflavin S staining of Aβ in three consecutive, 10 µm sections adjacent to the Visium spatial transcriptomics section. Sequential images were overlaid to reconstruct plaque locations and registered onto the original Visium image (scale bar = 1 mm).

bioRxiv preprint doi: https://doi.org/10.64898/2026.01.26.701799; this version posted January 28, 2026. The copyright holder for this preprint (which was not certified by peer review) is the author/funder, who has granted bioRxiv a license to display the preprint in perpetuity. It is made available under aCC-BY 4.0 International license.

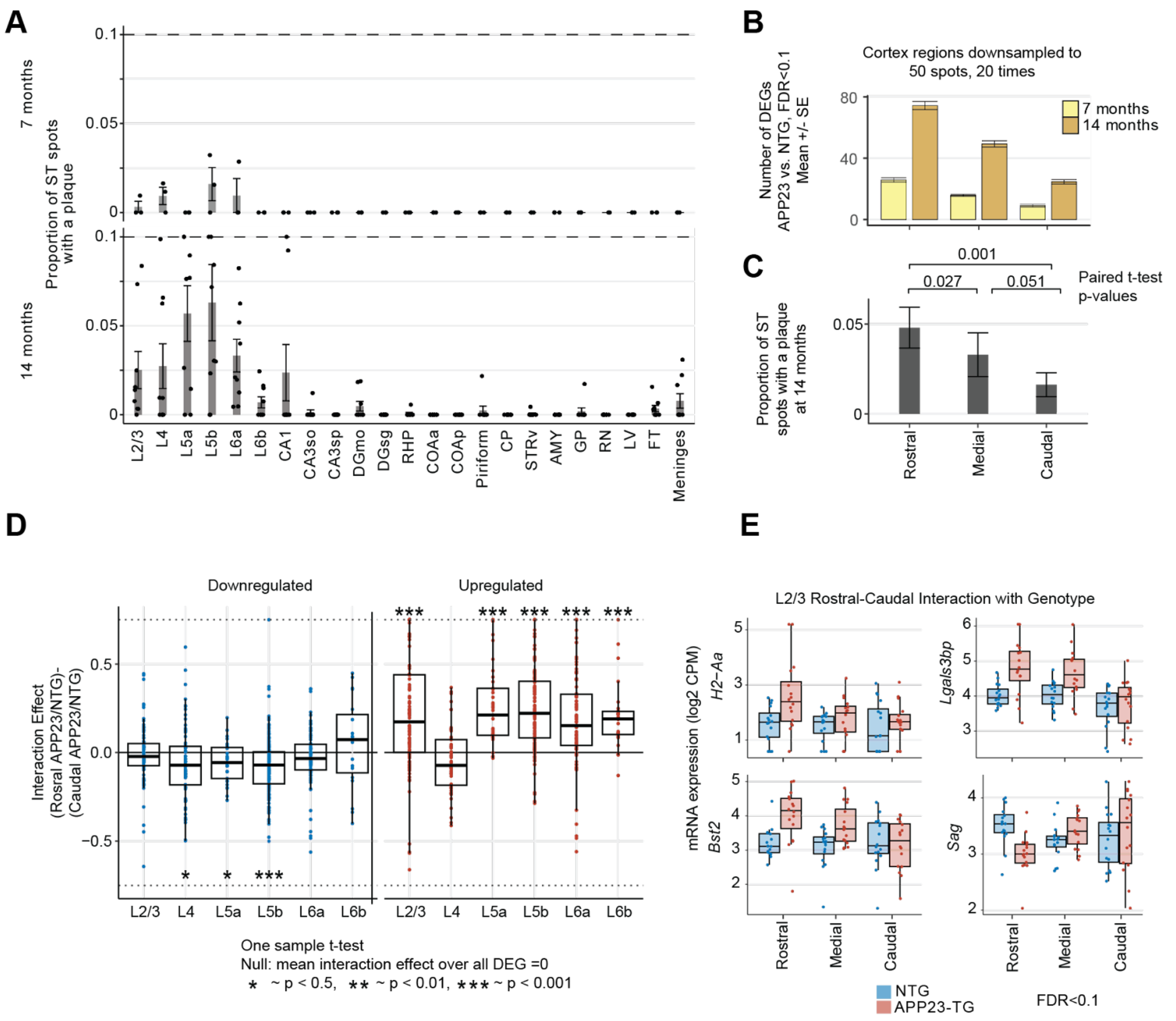

Figure S7: Regional plaque burden, differential expression and interaction effects in APP23-TG cortex. A, Proportion of ST spots containing amyloid plaques in APP23-TG mice at 7 and 14 months (n=16, 9). Bars and error bars show mean ± SE. **B,** Number of DEGs (DESeq2 Wald test FDR < 0.05) in APP23-TG versus NTG across cortical regions, computed on 20 50-spot down-samplings (mean ± SE). **C,** Proportion of plaque-positive spots at 14 months in APP23-TG samples (n=9, bars show mean ± SD); p-values from paired t-tests are shown. **D,** Regional modulation of the genotype effect on expression in each layer, shown separately for genes down-regulated (left) and up-regulated (right) in APP23-TG versus NTG. Each point is the per-gene difference in $\log_2$ fold-change between caudal and rostral cortex (i.e. regional shift of the genotype effect), boxplots summarize the distributions (horizontal line at zero indicates no regional difference), and one-sample t-tests evaluate whether the mean shift differs from zero (*p < 0.05; **p < 0.01; ***p < 0.001). **E,** Boxplots of gene expression for representative genes with significant interaction effects (FDR<0.1) between rostral and caudal cortex. Bars show mean ± SD.

bioRxiv preprint doi: https://doi.org/10.64898/2026.01.26.701799; this version posted January 28, 2026. The copyright holder for this preprint (which was not certified by peer review) is the author/funder, who has granted bioRxiv a license to display the preprint in perpetuity. It is made available under aCC-BY 4.0 International license.

Supplementary Table S1: Significantly rhythmic genes in all brain regions in non-transgenic (NTG) animals. Each tab shows DESeq2 results for one cluster, or the list of shared genes (FDR<0.1 in ≥10 regions). Columns include coefficients for covariates (age_7.months_vs_14.months, sex_M_vs_F) and for the sinusoidal rhythmic components (t_s,t_c); amplitude of the rhythmic component (log2_amplitude, where $amp = \sqrt{a^2 + b^2}$); acrophase is the phase of peak expression in ZT hours, $\phi = (24/2\pi)\ tan^{-1}\ (t_c/t_s)$.

Supplementary Table S2: Significantly rhythmic genes (FDR<0.05, DESeq2) in APP23-TG. Columns include the coefficients for covariates (age_7_months_vs_14months, sex_M_vs_F) and for the sinusoidal rhythmic components (a,b); phi is the phase of peak expression in radians, $\phi = tan^{-1}\ (b/a)\ mod\ 2\pi$, and phi_hr is the peak phase in ZT (hours), $\phi_{hr} = 24\phi/2\pi$; amp is the total amplitude of the rhythmic component ($amp = \sqrt{a^2 + b^2}$).

Supplementary Table S3: Differentially rhythmic genes (DRGs) between clusters (FDR<0.1). Tabs are separate analyses for NTG and APP23. Columns include the two regions being compared (test), labels for cluster 1 and cluster 2, test statistics (pvalue, padj from likelihood ratio test comparing models with separate sinusoidal components in the two regions vs. a single shared sinusoidal component), coefficients for covariates (mean cluster 1 vs. cluster 2) and for the sinusoidal rhythmic components for cluster 1, sinusoidal interaction terms for cluster 2, and derived rhythmic parameters per cluster (c1= cluster 1, c2=cluster2) as in table 3. For example, amplitude and phase for cluster 2: $amp_{c2} = \sqrt{(a + custer2.a)^2 + (b + cluster2.b)^2}, \phi_{c2} = tan^{-1} \frac{b+cluster2.b}{a+cluster2.a}\ mod\ 2\pi$

Supplementary Table S4: Differentially expressed genes (DEGs) in dorsal vs. ventral hippocampus (FDR<0.1). Each tab includes DEG results for one hippocampal cluster. Columns include gene, baseMean is the mean of DESeq2 size-factor normalized counts, log2FoldChange (dorsal vs. ventral), lfcSE is the standard error of the log2 fold change, stat is the Wald test statistic, p value (Wald test), and FDR_BH (Benjamini–Hochberg false discovery rate).

Supplementary Table S5: Differentially expressed genes (DEGs) between cortical regions. Each tab includes DEG results for one cortical layer. Columns include gene, contrast (region comparison e.g., RvI denotes rostral vs intermediate), baseMean is the mean of DESeq2 size-factor normalized counts, log2FoldChange, lfcSE is the standard error of the log2 fold change, stat is the Wald test statistic, p value (Wald test), and FDR_BH (Benjamini–Hochberg false discovery rate).

Supplementary Table S6: Cortex differentially rhythmic genes (DRGs) in rostral vs. caudal, intermediate vs. caudal, and rostral vs. intermediate regions. Each tab includes DRG results (FDR<0.1) for one comparison for each cortical cluster in NTG animals. Columns include gene, cluster, rhythmicity FDR in each region (rhythmicity_region_1/2), phase of peak expression in radians and hours (phi_region_1/2, phi_hr_region_1/2), and log2-scale amplitude in each region (amp_region_1/2).

bioRxiv preprint doi: https://doi.org/10.64898/2026.01.26.701799; this version posted January 28, 2026. The copyright holder for this preprint (which was not certified by peer review) is the author/funder, who has granted bioRxiv a license to display the preprint in perpetuity. It is made available under aCC-BY 4.0 International license.

Supplementary Table S7: KEGG pathway over-representation analysis for rostral-caudal DRGs. Enrichment analysis was performed for DRGs between rostral and caudal cortex for each layer of the cortex. DRGs with FDR<0.1 were split based on amplitude into rostral- or caudal biased and enrichment analysis was performed using the enrichKEGG function from clusterProfiler (*66*) using layer-specific rhythmic genes as background. Each tab includes results with adjusted p value<0.05 with columns specifying the cortical layer (cluster), KEGG pathway (Description), adjusted p value (p.adjust), and other default outputs from the enrichKegg function.

Supplementary Table S8: DEGs for APP23 vs NTG per cluster. Each tab includes DEGs between genotype for all clusters (FDR<0.1) for 7 months and 14 months of age. Columns include cluster, gene, baseMean (DESeq2 size-factor-normalized mean counts), log2FoldChange (APP23/NTG), lfcSE (standard error of the log2 fold change), stat (Wald test statistic), p value, and FDR_BH (Benjamini-Hochberg FDR). Note that Thy1 and humanAPP expression in the APP23 mice reflect the direct effect of the transgene.

Supplementary Table S9: Differentially expressed genes between Aβ plaque-associated spots and non-plaque associated. Results from a mixed effects model comparing gene expression in plaque-associated spots with non-plaque associated spots in the cortex (FDR<0.1, LRT: ~plaque+(1|sample) vs. (1|sample)). Columns include gene name, p-value, z score, adjusted p-value (padj) and log fold change (lfc).

Supplementary Table S10: DRGs for APP23 vs NTG per cluster. Each tab included DRGs with FDR<0.1 at 7 months and 14 months. Columns include cluster, gene, Intercept (APP23 mean), genotype_WT_vs_APP23 (NTG-APP23), sex_M_vs_F, t_s and t_c (APP23 sine and cosine terms), genotypeWT.t_s and genotypeWT.t_c (genotype by time interaction terms), log2-space amplitudes for APP23 and NTG (app_amp, wt_amp) and peak phase in radians and hours for APP23 and NTG (app_phi, app_phi_hr, wt_phi, wt_phi_hr), p value, and fdr_BH (Benjamini-Hochberg adjusted p values). Derived parameters follow the same logic as in Table 3, e.g $NTG\ amplitude = wt\_amp = \sqrt{(t_c + genotypeWT.tc)^2 + (t_s + genotypeWT.t_s)^2}$

Supplementary Table S11: ChEA3 transcription factor enrichment for cortical DRGs. Differentially rhythmic genes (FDR < 0.1) from all cortical clusters (APP23 vs. NTG at 7 months) were submitted to ChEA3 using the rChEA3 R package(*58*). Integrated meanRank and topRank results are filtered to transcription factors that were significant (FDR < 0.05) in at least one underlying ChEA3 library. For each individual library, transcription factors with library-level FDR < 0.05 are shown. For integrated results columns report transcription factor rank within the library (Rank), transcription factor symbol (TF), ChEA3 enrichment score (Score), the contributing ChEA3 library (Library), and the list of input genes overlapping each transcription factor target set (Overlapping_Genes). For individual libraries, column additionally report the number of overlapping genes between the input set and target set (Intersect), total size of the target set (Set length), Fisher's Exact Test p-value (FET p-value), library-level FDR, and the corresponding odds ratio.

bioRxiv preprint doi: https://doi.org/10.64898/2026.01.26.701799; this version posted January 28, 2026. The copyright holder for this preprint (which was not certified by peer review) is the author/funder, who has granted bioRxiv a license to display the preprint in perpetuity. It is made available under aCC-BY 4.0 International license.

(FDR<0.05) are highlighted red. **I,** Bubble plot of KEGG pathway enrichment of DRGs (APP23-TG vs. NTG).
